# Slow diffusive dynamics in a chaotic balanced neural network

**DOI:** 10.1371/journal.pcbi.1005505

**Published:** 2017-05-01

**Authors:** Nimrod Shaham, Yoram Burak

**Affiliations:** 1 Racah Institute of Physics, The Hebrew University of Jerusalem, Jerusalem, Israel; 2 Edmond and Lily Safra Center for Brain Sciences, The Hebrew University of Jerusalem, Jerusalem, Israel; Université Paris Descartes, Centre National de la Recherche Scientifique, FRANCE

## Abstract

It has been proposed that neural noise in the cortex arises from chaotic dynamics in the balanced state: in this model of cortical dynamics, the excitatory and inhibitory inputs to each neuron approximately cancel, and activity is driven by fluctuations of the synaptic inputs around their mean. It remains unclear whether neural networks in the balanced state can perform tasks that are highly sensitive to noise, such as storage of continuous parameters in working memory, while also accounting for the irregular behavior of single neurons. Here we show that continuous parameter working memory can be maintained in the balanced state, in a neural circuit with a simple network architecture. We show analytically that in the limit of an infinite network, the dynamics generated by this architecture are characterized by a continuous set of steady balanced states, allowing for the indefinite storage of a continuous parameter. In finite networks, we show that the chaotic noise drives diffusive motion along the approximate attractor, which gradually degrades the stored memory. We analyze the dynamics and show that the slow diffusive motion induces slowly decaying temporal cross correlations in the activity, which differ substantially from those previously described in the balanced state. We calculate the diffusivity, and show that it is inversely proportional to the system size. For large enough (but realistic) neural population sizes, and with suitable tuning of the network connections, the proposed balanced network can sustain continuous parameter values in memory over time scales larger by several orders of magnitude than the single neuron time scale.

## Author summary

This work studies the effects of chaotic dynamics, a prominent feature of the balanced state model, on storage of continuous parameters in working memory. We propose a simple model of a balanced network with mutual inhibition, and show that it possesses a continuum of steady states, a commonly proposed mechanism for maintenance of continuous parameter working memory in the brain. We use analytical methods, combined with large scale numerical simulations, to analyze the diffusive dynamics and correlation patterns generated by the chaotic nature of the system. We obtain new conclusions and predictions on irregular activity resulting from the combination of continuous attractor dynamics with the balanced state. These include a prediction of measurable, slowly decaying spike correlations, and a quantification of how persistence depends on the neural population size.

## Introduction

The consequences of irregular activity in the brain, and the mechanisms responsible for its emergence, are topics of fundamental interest in the study of brain function and dynamics. In theoretical models of brain activity, the irregular dynamics observed in neuronal activity are often modeled as arising from noisy inputs or from intrinsic noise in the dynamics of single neurons. However, theoretical and experimental works have suggested that explanations based on sources of noise in intrinsic neural dynamics are insufficient to account for the stochastic nature of activity in the cortex [1–4]. An alternative proposal is that noise in the cortex arises primarily from chaotic dynamics at the network level [3–6]. A central result in the field is that simple neural circuits with recurrent random connectivity can settle, under a broad range of conditions, into a fixed point called the balanced state [3, 7–9]: in this state the mean excitatory drive to each neuron nearly balances the mean inhibitory drive, and neural activity is driven by fluctuations in the excitatory and inhibitory inputs. The overall dynamics are chaotic, resulting in an apparent stochasticity in the activity of single neurons, which can exist even in the absence of any sources of random noise intrinsic to the dynamics of single neurons and synapses.

It remains unclear which computational functions in the brain are compatible with the architecture of the balanced network model, since this model assumes random, unstructured connectivity in its rudimentary form. The possibility that functional circuits in the brain are in a balanced state raises another important question: does the apparent stochasticity of single neurons in this state have similar consequences on brain function as would arise from stochasticity which is truly intrinsic to the neural and synaptic dynamics?

Here we explore the effects of chaotic noise on continuous parameter working memory. This task is particularly sensitive to noise, yet neurons in cortical areas involved in the maintenance of continuous parameter working memory have been shown to fire irregularly during task performance [10, 11]. Attractor dynamics are often put forward as a mechanism for the persistent neural activity underlying this task. Continuous attractor networks are dynamically characterized by a continuous manifold of semi-stable steady states, which make it possible to memorize parameters with a continuous range of values, such as an angle or a position [12–18]. In such networks, noise in neural or synaptic activity can cause diffusion along the manifold of steady states, leading to gradual degradation of the stored memory [19–21]. However, irregular activity in the balanced state does not arise from mechanisms intrinsic to neurons or synapses, but rather from chaotic dynamics, and its consequences for continuous parameter working memory are largely unexplored. For this reason we addressed two questions. First, we asked whether a neural network can possess a continuum of balanced stable states. Second, we investigated how, in this scenario, chaotic noise would affect information maintenance.

### Persistence in balanced networks

The question of whether balanced networks can produce persistent activity has attracted considerable interest in recent years. Several works explored architectures which give rise to slow dynamics in balanced networks, characterized by the coexistence of multiple discrete balanced states [22]. In several recent works multi-stability resulted from the existence of clustered connectivity, and slow transitions were observed between the discrete semi-stable states [23–25]. Other works [8, 26] demonstrated that a discrete set of semi-stable states can be embedded in a balanced neural network, using a similar construction as employed in the classical Hopfield model of associative memory [27].

A few works have addressed the possibility that balanced neural networks may generate slow persistent activity over a continuous manifold. Such dynamics were demonstrated in simulations of neural networks that included short-term synaptic plasticity [28], or a derivative-feedback mechanism [29, 30]. Previous works have not demonstrated the existence of a continuum of steady states in a balanced neural network analytically, and it has remained unclear whether such a continuum can be obtained without evoking additional mechanisms (such as short-term synaptic plasticity, or derivative-feedback). In addition, the influence of the chaotic dynamics on the persistence of stored memory has not been analyzed. These questions are addressed in the present work.

Below, we identify an architecture in which slow dynamics are attainable in a simple form of a balanced neural network. We prove analytically the existence of a continuous attractor in our model in the large population limit. In finite networks, we show that the chaotic noise drives diffusive motion along the attractor—leading, among other consequences, to slowly decaying spike cross-correlations. We show that the diffusivity scales inversely with the system size, as predicted previously for continuous attractor networks with intrinsic sources of neuronal stochasticity. With a reasonable number of neurons and suitable tuning, our model network exhibits slow dynamics over a continuous manifold of semi-stable states, while exhibiting single neural dynamics which appear stochastic, as observed in cortical circuits.

## Results

### Reciprocal inhibition between two balanced networks

Our neural network model is based on the classical balanced network model presented in Refs. [3, 7, 8]. This model consists of two distinct populations of binary neurons, one inhibitory and the other excitatory. The recurrent connectivity is random and sparse, with a probability *K*/*N* for a connection, where *N* is the population size (assumed for simplicity to be the same in both populations), *K* is the average number of connections per neuron from each population, and the connection strength is $∼1/\sqrt{K}$. For 1 ≪ *K* ≪ *N* and over a wide range of parameters, the mean population activity settles to a fixed point (the *balanced state*) where on average the total excitation received by each neuron is approximately canceled by the total inhibition (to leading order in $1/\sqrt{K}$), and the neural dynamics are driven by the fluctuations in the input. The single neuron activity appears noisy, neither of the populations is fully activated or deactivated, and the overall network state is chaotic.

**Fig 1:**
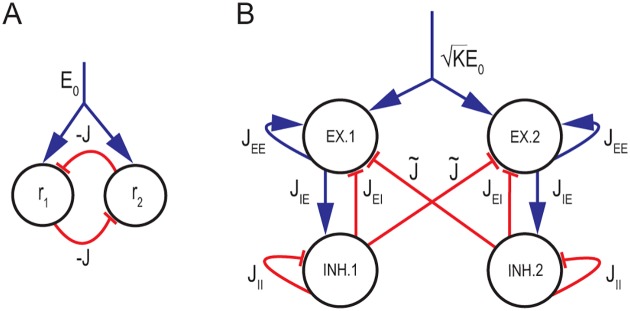
Network architecture and parameters. **A** Two neural populations with rates *r*_1_, *r*_2_ inhibit each other with synaptic efficacies −*J*. **B** Two coupled balanced subnetworks, each consisting of an excitatory and inhibitory population. Excitatory (inhibitory) connections are represented in blue (red). Mutual inhibition is generated by all-to-all connections of strength $-\tilde{J}\sqrt{K}/N$ from each inhibitory population to the excitatory population of the other subnetwork. As in [7], connections within each subnetwork are random with a connection probability *K*/*N*, 1 ≪ *K* ≪ *N*. Connection strengths are: $J_{EE}/\sqrt{K},\;J_{IE}/\sqrt{K},\;J_{EI}/\sqrt{K}$ and $J_{II}/\sqrt{K}$ according to the identity of the participating neurons. Without loss of generality, we chose *J*_*EE*_ = *J*_*IE*_ = 1 and define *J*_*EI*_ ≡ −*J*_*E*_, *J*_*II*_ ≡ −*J*_*I*_. An excitatory input $\sqrt{K}E_{0}$ is fed into both excitatory populations.

Despite the nonlinearities involved in the dynamics of each neuron, the population averaged activities in the balanced state are linear functions of the external input [3, 7]. We exploit this linearity to build a simple system of two balanced networks projecting to each other. The intuition comes from a simple model of a continuous attractor neural network consisting of linear neurons arranged in two populations that mutually inhibit each other, Fig 1A. The linear rate dynamics of this system are given by:

$$\begin{array}{c} \tau \dot{r}=-r+Wr+E\;, \end{array}$$ (1)

where ***E*** = [*E*_0_, *E*_0_], *E*_0_ > 0 is an external input and

$$\begin{array}{c} W=\left(\begin{array}{cc} 0 & -J\\ -J & 0 \end{array}\right)\;. \end{array}$$ (2)

For *J* = 1 the system has a vanishing eigenvalue, and the fixed points form a continuous line: *r*_1_+*r*_2_ = *E*_0_.

The simple neural architecture of Fig 1A was used as a basis for modeling the dynamics of neural circuits responsible for memory and decision making in the prefrontal cortex [31–35]. In our model, a balanced subnetwork replaces each of the populations of Fig 1A, and the inhibitory population in each subnetwork projects to the excitatory population of the other subnetwork, Fig 1B and *Methods*. Thus, the model consists of two reciprocally inhibiting balanced neural populations.

We consider first a scenario in which the inhibitory connectivity between the two sub-networks is all-to-all. Therefore, the network includes a combination of strong, random synapses within each sub-network and highly structured, weak synapses between the two sub-networks. This scenario lends itself to analytical treatment of finite *N* effects (see below). Later on, we present results also for an alternative scenario, in which the connections between the two-subnetworks are sparse, random, and strong (*Additional randomness in connectivity and inputs*).

### Continuum of balanced states

We first examine whether the two-subnetwork architecture can give rise to a continuum of balanced states. The parameters of the network connectivity in our model are summarized in Fig 1 and in *Methods*. The mutual inhibition between the subnetworks is assumed to be all to all, and the interaction strength is scaled such that the total inhibitory input to each neuron, coming from the opposing subnetwork scales in proportion to $\sqrt{K}$.

Similar to the case of a single balanced network [7], the mean field dynamics of the population averaged activities for *N* → ∞ and *K* ≫ 1 are given by:

$$\begin{array}{c} \tau_{i}{\dot{m}}_{i}=-m_{i}+H\left(-u_{i}/\sqrt{\alpha_{i}}\right)\;, \end{array}$$ (3)

where $m_{i}\left(t\right)=1/N\sum_{k=1}^{N}\sigma_{i}^{k}\left(t\right)$ [*i* = 1 (2) for the excitatory (inhibitory) population of the first subnetwork, and similarly *i* = 3, 4 in the second subnetwork], $\sigma_{i}^{k}\left(t\right)$ is the state of neuron *k* in population *i* at time *t*, *H*(*x*) is the complementary error function, and *u*_*i*_ (*α*_*i*_) is the mean (variance) of the input to a neuron in population *i*, averaged over the population and over the random connectivity (Methods). This equation is an approximation which becomes exact in the limit *K* → ∞.

To check whether there exist parameters for which the system has a continuum of balanced states, it is convenient to write the steady state equations of the above dynamics, while making use of the assumption that *K* is large. In the limit *K* → ∞ these equations become linear (Methods):

$$\begin{array}{c} \begin{array}{ccc} m_{1}-J_{E}m_{2}-\tilde{J}m_{4}+E_{0} & = & 0\;,\\ m_{1}-J_{I}m_{2} & = & 0\;,\\ m_{3}-J_{E}m_{4}-\tilde{J}m_{2}+E_{0} & = & 0\;,\\ m_{3}-J_{I}m_{4} & = & 0\;. \end{array} \end{array}$$ (4)

By choosing the interaction strength between the two subnetworks to be $\tilde{J}=J_{E}-J_{I}$, this system becomes singular, and has a continuum of solutions arranged on a line in the mean activities space, which represent a continuum of stable balanced states.

#### Finite *K*

When *K* is large and finite, but still in the idealized limit of infinite *N*, the population dynamics remain deterministic, and are approximately described by Eq 3, but the nullclines corresponding to the steady state equations (Eq 13) are now nonlinear. Therefore, a precise continuum of steady states cannot be established. However, if the nonlinear nullclines are close to each other, slow dynamics are attainable in a specific direction of the mean activity space. To make this statement more precise, we first note that the steady state equations always have a symmetric solution in which *m*_1_ = *m*_3_ and *m*_2_ = *m*_4_. If in addition, at this symmetric point, the slopes of the nullclines are identical (∂*m*_3_/∂*m*_1_ = −1), there is a vanishing eigenvalue of the linearized mean field dynamics (Eq 3) around this point (Methods).

**Fig 2:**
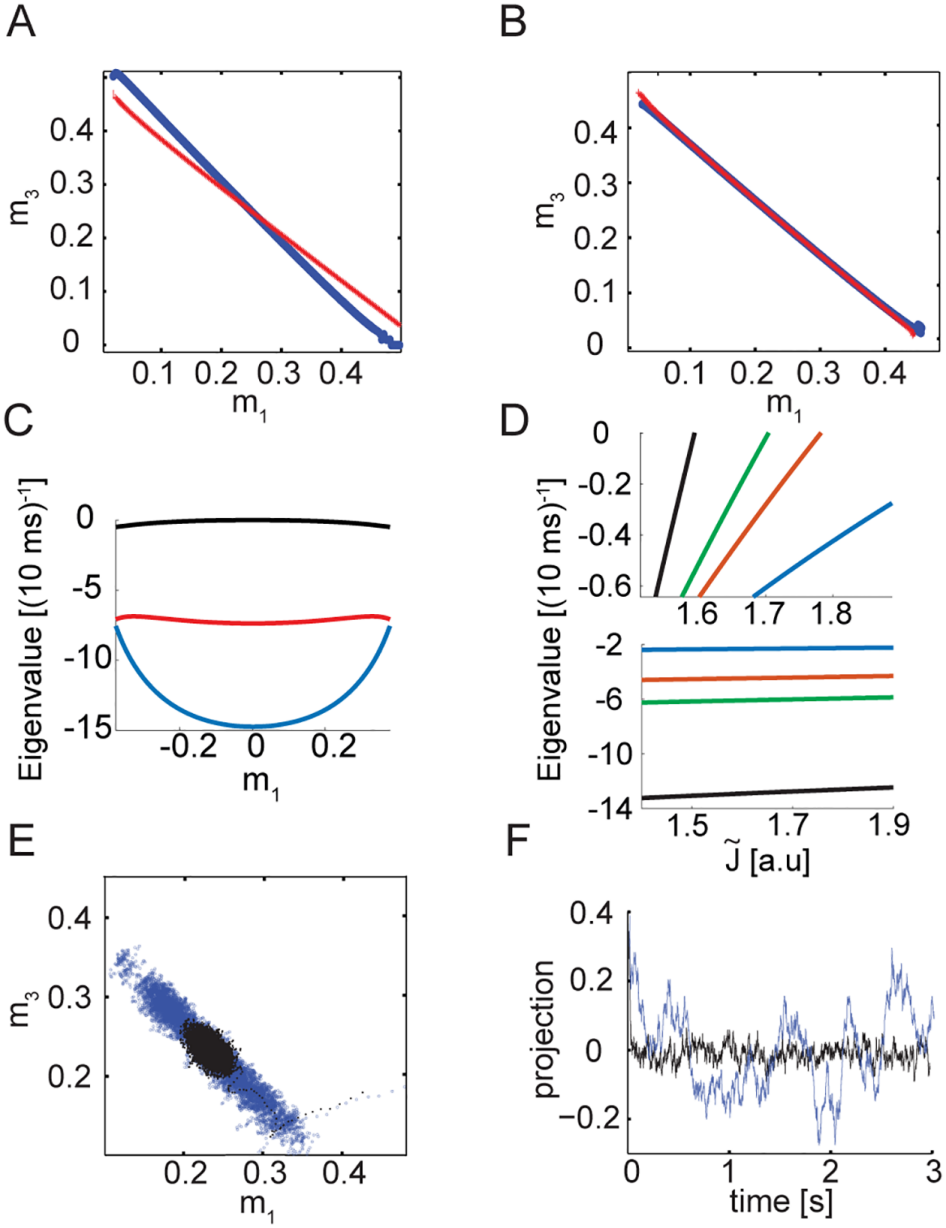
Dynamics in the *N* → ∞ limit. **A** Projections of the nullclines ${\dot{m}}_{1}=0$ (blue) and ${\dot{m}}_{3}=0$ (red) on the *m*_1_ − *m*_3_ plane, based on Eq 13. Here *K* = 1000, *J_E_* = 4, *J_I_* = 2.5, $\tilde{J}=1.5$, *τ_E_* = 10 ms, *τ_I_* = 8 ms, and *E*_0_ = 0.3. The same values of *J*_*E*_, *J*_*I*_, *E*_0_, *τ*_*E*_, and *τ*_*I*_ are used throughout the manuscript. **B** Same as A, except that here $\tilde{J}≃1.7$, tuned to achieve a singular Jacobian at the symmetric point. **C** Real part of the four eigenvalues of the Jacobian evaluated at different points along the approximate attractor (here parametrized by the value of *m*_1_). Note that there are two complex conjugate eigenvalues, and their real parts overlap (red curve). **D** Top: the eigenvalue closest to zero as a function of the mutual inhibition strength. Different colors correspond to different values of *K*: 5000 (black), 1000 (green), 500 (red) and 100 (blue). All other parameters are as in A. Bottom: real part of the eigenvalue next to closest to zero as a function of the mutual inhibition strength. **E** Integration of Eq 3 with injected uncorrelated Gaussian noise with $\sigma =10^{-2}\;\sqrt{10\text{msec}}$ (Eqs 15 and 16), $\tilde{J}=1.5$ (black), $\tilde{J}≃1.7$ (blue). **F** Dynamics of the projection along the special direction (parameters and colors as in E).

Under these conditions, the smallest eigenvalue of the linearized dynamics is expected to be small also in the vicinity of the symmetric point. In fact, even for moderately large values of *K* (*K* = 1000), the two nullclines nearly overlap over a large range of *m*_1_ and *m*_3_, Fig 2A and 2B. Fig 2C demonstrates that in this case there is an eigenvalue close to zero within a wide range of locations along the approximate attractor, and therefore the dynamics are slow at any position along this range. Below, we denote by *λ* the eigenvalue closest to zero of the linearized dynamics, evaluated at the symmetric fixed point.

As observed in other continuous attractor neural network models, the slow dynamics are sensitive to the tuning of the recurrent connectivity [12, 15, 29, 33] (see also Discussion). This sensitivity is quantified by the dependence of *λ* on the coupling strength between the two subnetworks. Fig 2D (top panel) shows how *λ* depends on the mutual inhibition strength $\tilde{J}$ and on *K*: *λ* is linear in $\tilde{J}$, and proportional to $\sqrt{K}$. For *K* = 1000, $\tilde{J}$ must be tuned to a precision of ∼0.1% to achieve a time scale *λ*^−1^ of several seconds, when the intrinsic time scale *τ* is 10 ms. The real part of the next eigenvalue is negative, proportional to $\sqrt{K}$, and is weakly dependent on $\tilde{J}$ (Fig 2D, bottom panel).

We find that the approximate line attractor is stable to small perturbations over a wide range of parameters. This is verified by observing that when linearizing the population dynamics (Eq 3) around positions along the approximate attractor, the real parts of the four eigenvalues are negative, and one of them is close to zero, reflecting the slow dynamics along the approximate attractor—as demonstrated in Fig 2C.

As an illustration of the existence of a direction in mean activity space, along which the dynamics are slow, we numerically solved the mean field differential equations in the limit of infinite *N* and *K* = 1000, with injected white noise. Fig 2(E) and 2(F) shows that the resulting mean activities trace a line in the mean activities space (E) and the dynamics along the line are slow (F).

### Diffusive dynamics in finite size networks

Next, we consider the realistic situation in which *N* is finite in the two-subnetwork model, while still requiring that *N* ≫ *K* ≫ 1. Instead of adding noise to the dynamics of each neuron, we ask whether the chaotic dynamics are sufficient to drive diffusive motion along the approximate attractor. This question is motivated by the fact that diffusive dynamics are observed in model neural networks of intrinsically noisy neurons, with a finite number of neurons [19]. In addition, this question is motivated by evidence of diffusive dynamics underlying continuous parameter working tasks—as observed both in the behavioral data and in its neural correlates in the prefrontal cortex [10, 11, 36].

Since the population dynamics are no longer given by Eq 3, we performed large scale numerical simulations of networks with *N* ranging between 10^4^ to 15 × 10^4^ (additional details on the simulations can be found in *Methods*). To simplify the analysis and the presentation, we chose the random weights within each subnetwork such that they precisely mirrored each other, which ensured that the fixed point would be symmetric (*m*_1_ = *m*_3_ and *m*_2_ = *m*_4_). If, alternatively, the connections in each subnetwork are chosen independently, the fixed point deviates slightly from this symmetry plane (this deviation approaches zero for infinite networks). However, all the results described below remain qualitatively valid (see below, *Additional randomness in connectivity and inputs*).

**Fig 3:**
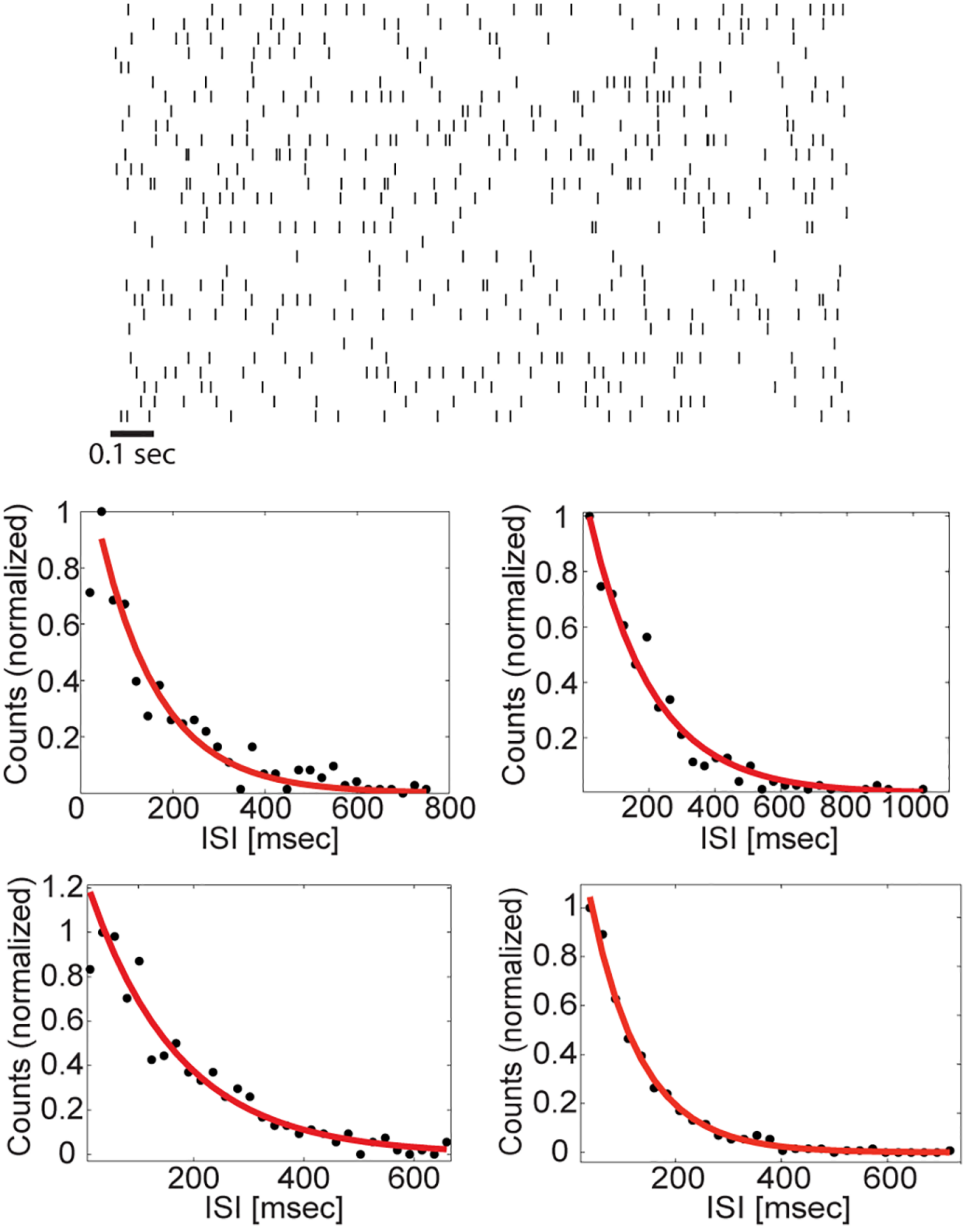
Single neuron statistics. Top: Raster plot of 30 neurons from one of the excitatory populations in resting state. Here a spike is defined as a transition from an ‘off’ state to an ‘on’ state. Bottom: inter-spike interval distribution of four representative neurons from one of the excitatory populations. A fit to an exponential function is shown as a solid red line.

**Fig 4:**
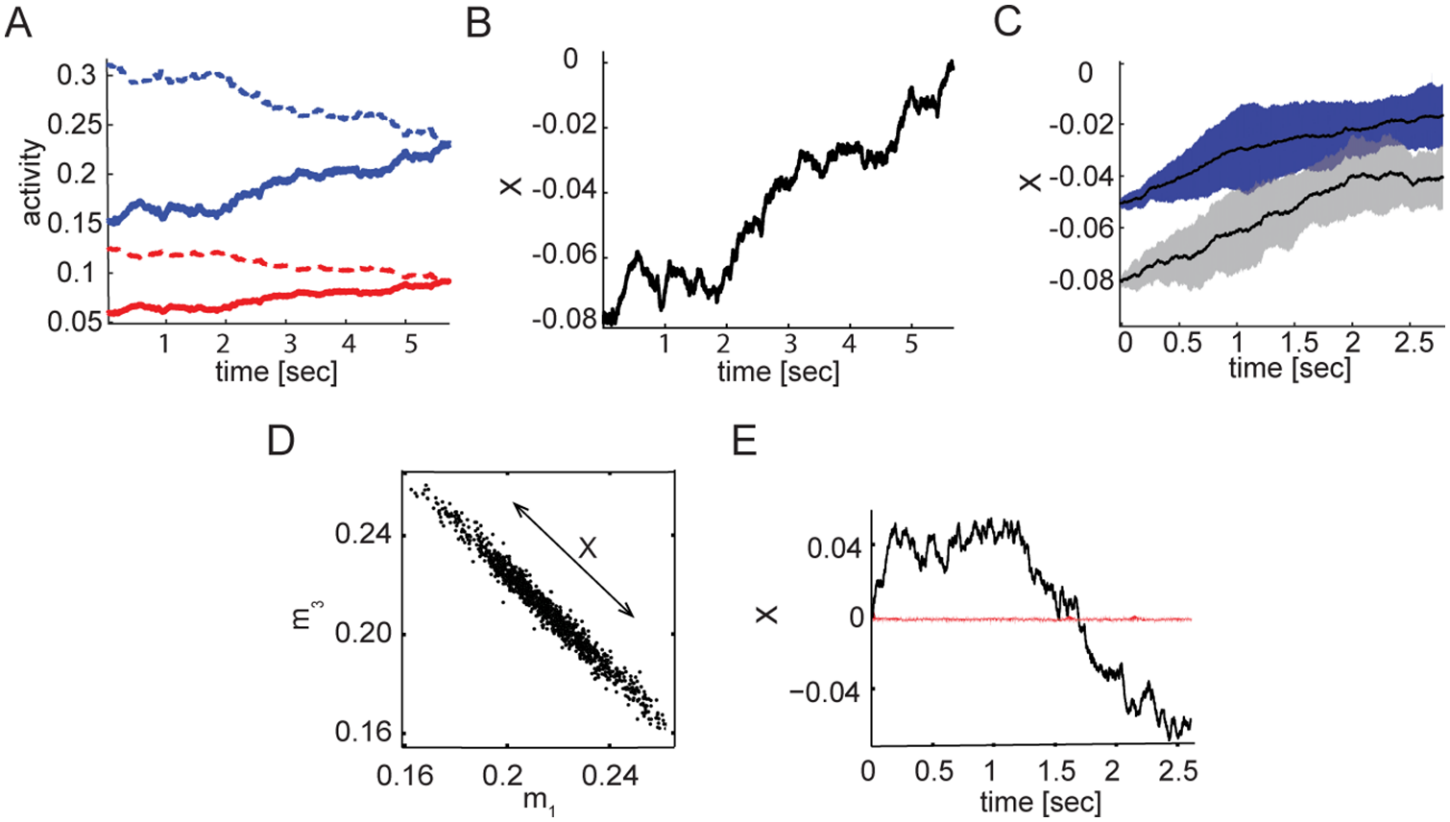
Dynamics of finite *N* networks. **A** Mean activities of the four populations after initialization at a specific state along the approximate attractor. Blue (red) traces are used for the excitatory (inhibitory) populations. Dashed and solid lines are used to distinguish between the two subnetworks. **B** Projection along the approximate attractor, shown for the same simulation as in A. **C** Projected dynamics for two initial conditions: *X*(0) = 0.05 (blue shaded area) and *X*(0) = 0.08 (grey shaded area). Black lines are averages over 50 trials. Shaded areas represent the standard deviation measured over 50 independent trials. **D** Projection of the mean activities on the *m*_1_-*m*_3_ plane in resting state activity. **E** Dynamics of the projection *X* along the special direction (black), and a projection on a perpendicular direction (red) in the same simulation as in D. In A, B and C *N* = 1.5 × 10^5^, *K* = 500 and $\tilde{J}≃1.77$. In D and E *N* = 10^5^, *K* = 1000 and $\tilde{J}≃1.69$. Other parameters are as in Fig 2.

The neural activity observed in our simulations is irregular and individual neurons approximately exhibit exponential ISI distributions similar to those observed in the two population case, although their dynamics are deterministic (Fig 3). To test whether the network can perform short term memory tasks, we initiated the population activities such that the network state was close to some point along the approximate line attractor. Fig 4A shows the resulting dynamics of the four populations: the activities persisted for a few seconds before decaying towards the symmetric fixed point. Fig 4B shows the projection along the slow direction, *X*(*t*) (defined in Eq 17), again revealing the slow decay of the initial state. Fig 4C shows statistics of trajectories that start from two initial positions along the approximate attractor, when $\tilde{J}$ is tuned to achieve λ^−1^ ≃ 9 *s*. The state of the network enables discrimination between the two conditions over a time scale of several seconds. The ability to do so with high confidence is influenced both by λ and the stochasticity of the motion, which we characterize in the following section (see also Discussion). S1 Fig shows the mean square displacement (MSD) from the starting point for the same dataset, averaged over all trials.

Long after initialization, the population activities fluctuate around the symmetric fixed point, along a line corresponding to the approximate attractor: a projection on the *m*_1_ − *m*_3_ plane is shown in Fig 4D. Fig 4E demonstrates that *X*(*t*) exhibits slow diffusive dynamics. To demonstrate that the dynamics are effectively one dimensional, a projection on a perpendicular direction is shown as well.

#### Statistics of the diffusive motion

From here on we focus on the dynamics of *X*(*t*), the projected position along the approximate attractor. The dynamics of *X* can be characterized by the two moments:

$$F\left(X,\Delta t\right)\equiv {\langle X\left(t+\Delta t\right)-X\left(t\right)|X\left(t\right)=X\rangle }_{t},$$ (5)

$$G\left(X,\Delta t\right)\equiv {\langle {\left[X\left(t+\Delta t\right)-X\left(t\right)\right]}^{2}|X\left(t\right)=X\rangle }_{t}.$$ (6)

**Fig 5:**
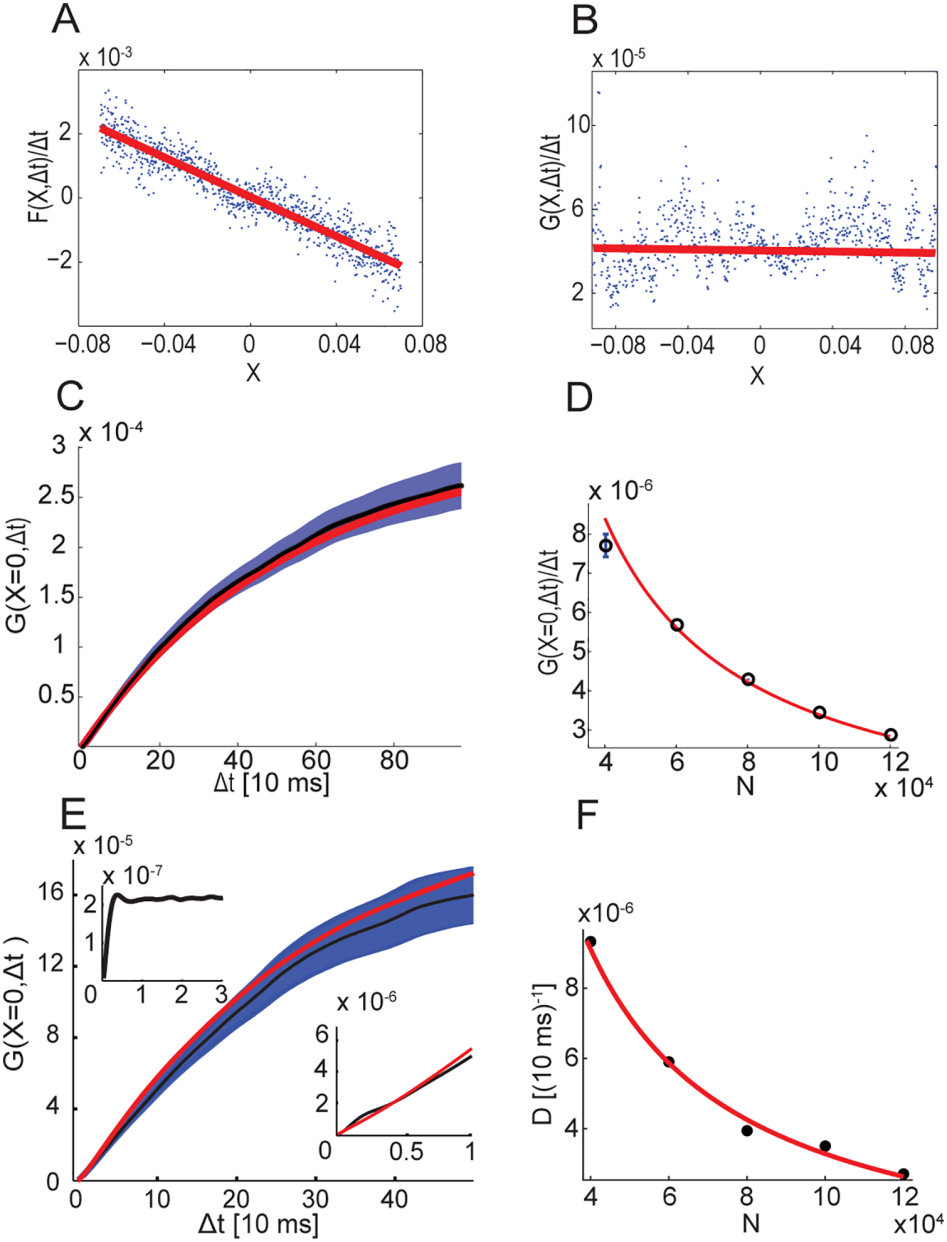
Statistics of motion along the approximate attractor. **A**-**B** Statistics of motion along the attractor over short time scales: mean rate of drift *F*(*X*, Δ*t* → 0)/Δ*t* (A), and random diffusion *G*(*X*, Δ*t* → 0)/Δ*t* (B), measured in numerical simulations as a function of the location *X* along the approximate attractor. A linear fit is shown in red in both panels. Here *N* = 30000, *K* = 1000, Δ*t* ≃ 3 ms. **C** Numerical measurement of *G*(*X* = 0, Δ*t*) (Black trace. The shaded blue area designates the standard deviation of the mean) and a fit to the statistics of an OU process (red). **D** Measurement of *G*(*X*, Δ*t* → 0) (black rings, error bars are smaller than the rings if not shown), compared with the analytical expression, Eq 28 (red trace). Here *N* = 1.2 × 10^5^. **E** Measurements of *G*(*X*, Δ*t*) from simulations (Black trace. The shaded blue area designates the standard deviation of the mean, same as in C), compared with the semi-analytical approximation (red), Eq 36 (*N* = 1.2 × 10^5^). Lower inset: zoom-in on Δ*t* ≤ *τ*. Upper inset: measurement of *G*(*X* = 0, Δ*t*) from a single balanced network. Here we chose *X* = *m*_1_(*t*) − 〈*m*_1_(*t*)〉_*t*_. **F** Diffusion coefficient (extracted from a fit to an OU process), shown as a function of *N*. Symbols: simulations, red trace: fit to ∼1/*N* dependence.

The first moment *F* characterizes the systematic component of drift along the approximate attractor, and the second moment *G* characterizes the random, diffusive component of the motion. Both moments may depend, in general, on the position *X* along the approximate attractor. Fig 5(A) and 5(B) show measurements from simulations of *F*(*X*, Δ*t*) and *G*(*X*, Δ*t*) in the limit of small Δ*t*, at various locations *X*.

For small Δ*t* and near the fixed point, we expect *F*(*X*, Δ*t*) ≃ − *λXΔt* with constant *λ*, where in the limit of large *N* and *K*, *λ* becomes equal to the smallest eigenvalue of the deterministic linearized dynamics at the symmetric fixed point. In fact, this relation holds to a very good approximation over a wide range of positions along the approximate attractor (Fig 5A).

The moment *G*(*X*, Δ*t*) (Eq 6) characterizes the diffusion along the approximate line attractor, and is the focus of our analysis in the rest of this section, since it quantifies the random aspect of the dynamics, driven by the chaotic noise. Fig 5B shows measurements of this quantity from simulations (for small Δ*t*), over a wide range of positions along the approximate attractor. Note that for the parameters we use and our choice for the parametrization of *X* (Methods), the range of *X* is approximately [−0.2, 0.2]. Fig 5C shows measurements of *G*(*X*, Δ*t*) for *X* = 0, over a wide range of time intervals.

#### Short time scales

Our main interest lies in the diffusive motion over long time scales compared to *τ*. However, we consider first the diffusive motion over short time scales, quantified by *G*(*X*, Δ*t*) for Δ*t* ≲ *τ*, since in this case the behavior of *G* can be expressed exactly in terms of the averaged autocorrelation function, $q_{j}\left(\Delta t\right)\equiv 1/N\sum_{i=1}^{N}\langle \sigma_{i}^{j}\left(t+\Delta t\right)\sigma_{i}^{j}\left(t\right)\rangle$ (Methods). Using the mean field theory, it is possible to derive a differential equation for *q*(*t*) [7], which can be solved numerically. Using the numerical solution, we obtained a prediction for *G*(*X*, Δ*t*) which is in excellent agreement with measurements from numerical simulations, Fig 5D. Note that there are no fitting parameters in this calculation.

This analysis leads to two conclusions, which are important for the analysis that follows below: first, *G* is proportional to Δ*t* for small Δ*t*. Second, *G* is inversely proportional to *N*, the size of the neural populations. A similar derivation can be applied also to the single balanced network discussed in [7], for Δ*t* ≪ *τ* (upper inset in Fig 5E).

In addition, we note that *G*(*X*, Δ*t* → 0)/Δ*t* is approximately constant along the approximate attractor, as seen in Fig 5B. Therefore, in most of the numerical results below we focus on *G* near the symmetric fixed point (*X* = 0).

#### Diffusion over arbitrary time scales

On time scales larger than *τ*, the behavior of the two coupled balanced subnetworks differs dramatically from that of the single balanced network: in the single balanced network *G* saturates for Δ*t* ≳ *τ* (Fig 5E, upper inset), whereas in the two coupled balanced subnetworks *G* continues to increase as a function of Δ*t*, up to Δ*t* of order *λ*^−1^ (Fig 5E, main plot). Thus, the diffusive motion generates correlated activity over time scales much longer than *τ*. Because the chaotic noise itself is uncorrelated on time scales longer than *τ* (as shown more precisely below), and since *λ* is approximately constant along the approximate attractor, we may expect the motion to approximately follow the statistics of an Ornstein–Uhlenbeck (OU) process. This approximation provides a good fit to the dynamics, Fig 5C, as expected. This made it possible to extract a diffusion coefficient *D* from the simulations which characterizes the random motion on time scales *τ* ≲ Δ*t* ≲ *λ*^-1^. Furthermore, since *D* and *λ* are approximately constant over a wide range of positions along the approximate attractor (Fig 5A and 5B), the approximation as an OU process provides a precise and compact description of the trajectory statistics, from which the performance of the network in retention of memory can be deduced (see Discussion and Fig 5C, S1, S2D, S3E and S4B and S4C Figs).

According to Eq 28 (Methods), fluctuations in the mean activity scale as 1/*N*, but this equation is valid only for time scales smaller than *τ*, whereas the diffusion coefficient *D* characterizes fluctuations on longer time scales. Fig 5F demonstrates that the 1/*N* scaling holds also for the diffusive motion over long time scales: the diffusion coefficient, extracted from a fit to the statistics of an OU process, is inversely proportional to *N*. The same scaling with *N* has been observed in continuous attractor networks with intrinsic neural stochasticity [19]. Another important implication of the 1/*N* scaling is that sufficiently large networks can reliably store a continuous variable in short-term memory (see Discussion).

**Fig 6:**
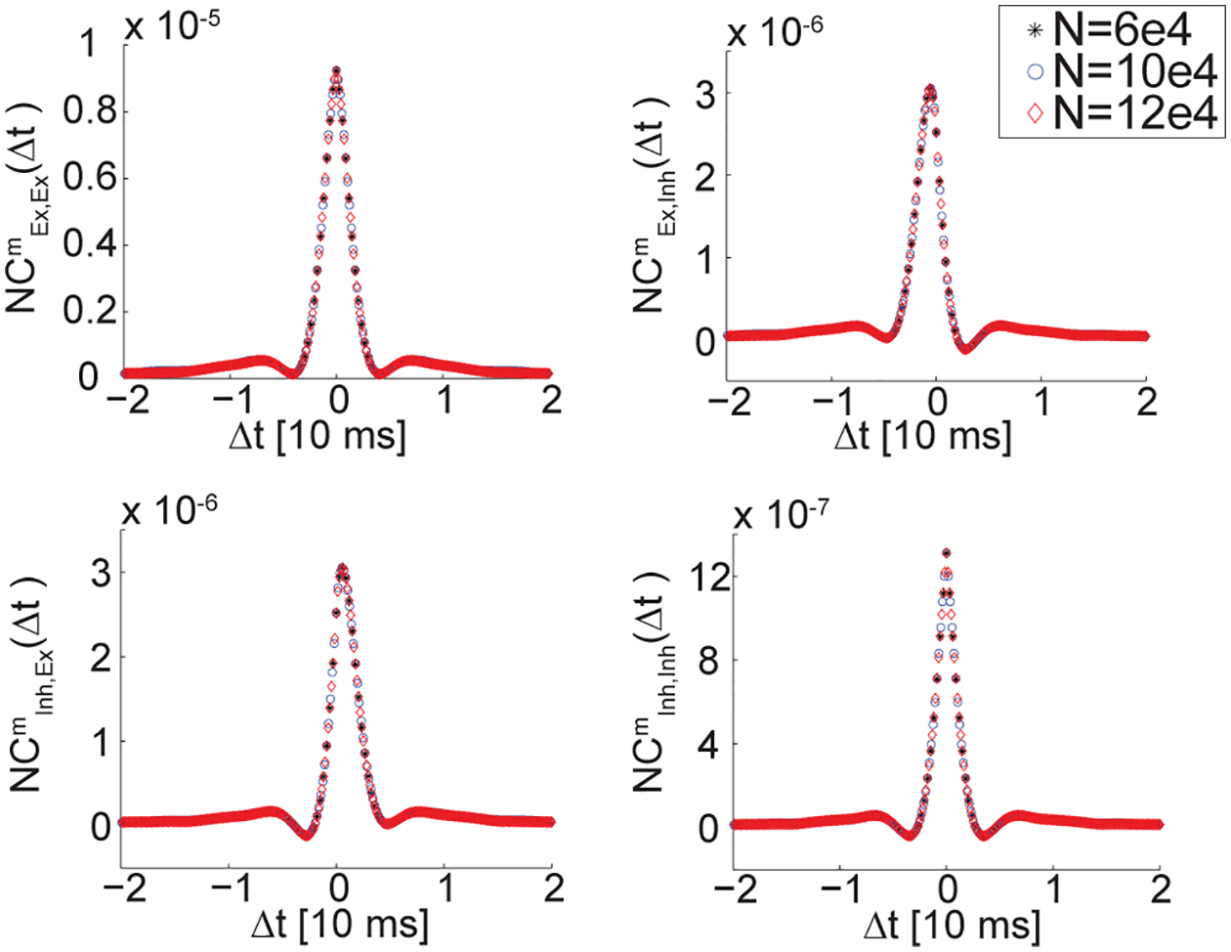
Cross correlations in a single balanced network. Population averaged cross-correlations of neural activity in a single balanced network, *C*^*m*^(Δ*t*) (Eq 7), shown as a function of the time lag Δ*t* (in units of *τ* = 10ms). The cross-correlation functions are multiplied by *N*, to demonstrate that *C*_*ij*_ scale as 1/*N*: with this choice of scaling, measurements from simulations with different values of N collapse on one curve.

To understand this result in more detail, we start by considering the time dependent correlation functions of *m*_*i*_ in a *single* balanced network:

$$\begin{array}{c} C_{ij}^{m}\left(\Delta t\right)\equiv \frac{1}{N^{2}}\sum_{k,l=1}^{N}\left[{\left\langle \sigma_{i}^{k}\left(t+\Delta t\right)\sigma_{j}^{l}\left(t\right)\right\rangle }_{t}-{\left\langle \sigma_{i}^{k}\left(t+\Delta t\right)\right\rangle }_{t}{\left\langle \sigma_{j}^{l}\left(t\right)\right\rangle }_{t}\right]\;, \end{array}$$ (7)

where *i*, *j* ∈ {Ex,Inh}. An analytical expression for these correlation functions is not available (see [9] for further discussion). Therefore, we measured them numerically in simulations of activity in the single balanced network architecture. These measurements indicate that the time-dependent correlation functions decay over time scales of order *τ*, and that they scale as 1/*N*, Fig 6.

Next, we show that the statistics of diffusion in the coupled system can be expressed precisely in terms of the correlation functions of the single, uncoupled balanced networks (Methods). Thus, the correlation structure of the chaotic noise in the single balanced network determines the statistics of the slow diffusive motion along the approximate attractor in the coupled two-population network.

Using the noise cross correlations measured in simulations of a single balanced network, it is possible to obtain a semi analytical approximation for *G* in the system of two coupled subnetworks, which does not involve any fitting parameters. The measurements of *G* from simulations are in excellent agreement with this analytical prediction, Fig 5E. The above analysis indicates that the 1/*N* scaling of the diffusion coefficient (Fig 5F) is a consequence of the decay with *N* of cross correlations in activity of different neurons in the single balanced network. In this sense, for large *N* the network behaves as a collection of neurons with independent random noise, although the source of this apparent noise is the chaotic activity generated by the recurrent connectivity.

### Spike correlation functions

**Fig 7:**
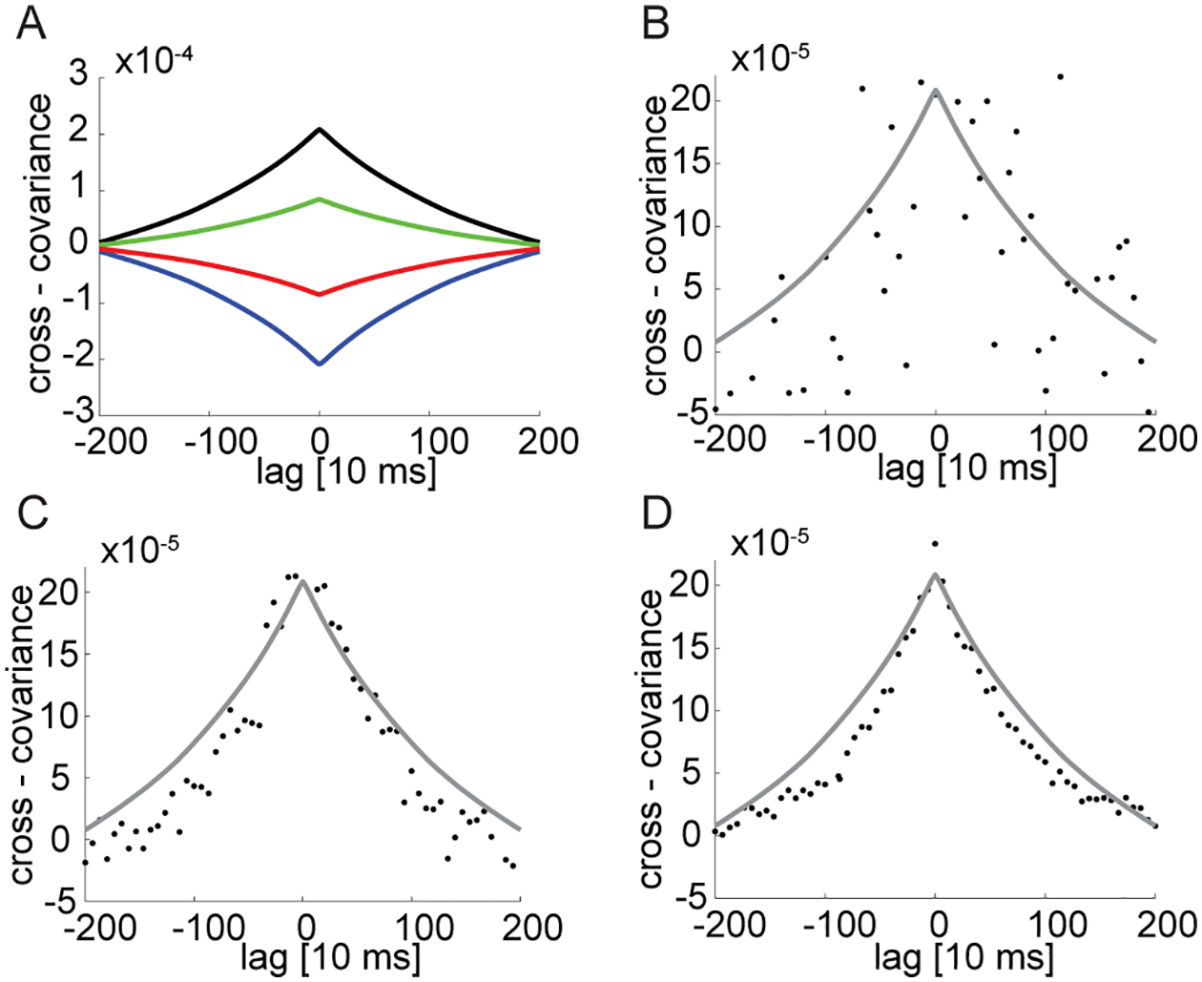
Cross correlations in the coupled subnetwork architecture. **A** Auto-covariance of the mean activity of one of the excitatory populations (black). Cross covariance between the first excitatory population and the first inhibitory population (green), the second inhibitory population (red) and the second excitatory population (blue). **B**-**D** Pairwise correlation of neuron activity in one of the excitatory populations. Correlations are measured in *n* neurons, selected randomly, and averaged over all pairs (Methods), where *n* = 10 (B), 50 (C), and 100 (D). The simulated time is 15 minutes. An average over the entire population is shown in grey. In all panels *N* = 1.2 × 10^5^, *K* = 1000, *λ*^−1^ ≃ 2 sec.

The diffusion along the approximate attractor implies that the population activities are correlated over long time scales, up to order *λ*^−1^: Fig 7A shows examples of the population correlation functions *C*^*m*^, which differ dramatically from those of the single balanced network, Fig 6 (note the different time scales in the two sets of figures).

Spike trains generated by single neuron pairs are correlated over long time scales as well, since all neurons in the network are coupled to the collective diffusion along the approximate attractor. However, a reliable observation of the slowly decaying correlation in a single pair might require an unrealistically long recording time. This difficulty can be overcome potentially by considering the simultaneous activity of multiple neurons: for example, we find in our simulations of a network with *N* = 10^5^ that for 15 minutes of simulated time, a simultaneous recording from ∼50 or more neurons from each population would be sufficient to reliably observe the slow temporal decay of the correlations, Fig 7C, whereas a simultaneous recording from ten neurons over 15 minutes may be insufficient. As demonstrated in Fig 7(B)–7(D) the noise falls as one over the number of measured neurons and as one over the total recording time. Hence, by extrapolating from the results in Fig 7(B)–7(D), ∼12 hours of recording would be required to obtain a measurable correlation signal from a single pair of neurons.

### Chaotic behavior

**Fig 8:**
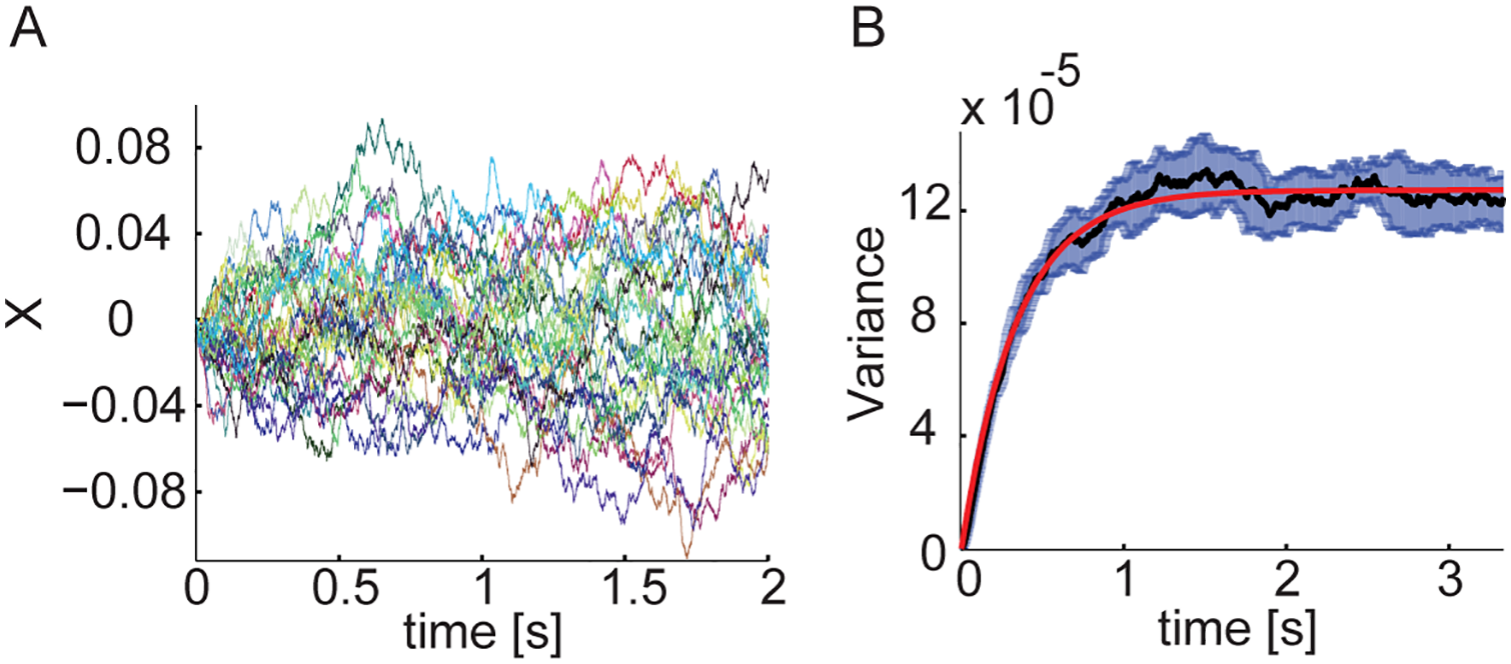
Chaotic nature of the noise driving the diffusive motion. **A** Projection *X* of the mean activities on the approximate attractor in 30 trials with the same update schedule and the same initial conditions, except for one neuron which was flipped in each population (*N* = 10^5^). **B** Variance over 1500 trials as a function of time, with 2*σ* error bars (gray). Red: fit to the variance of an OU process (*D* ≃ 3.4 ⋅ 10^−6^1/10s).

Next, we briefly address the chaotic nature of the noise that drives diffusive motion. Fig 8A shows results from multiple simulations in which the initial network state differed solely by a flip of one neuron in each population (out of ∼10^5^ neurons). All other parameters, including the asynchronous update schedule and the network weights were identical across runs. The time dependence of the variance across different runs is similar to the variance over realizations of an OU process, Fig 8B, with a similar diffusion coefficient as observed in the fit for *G*(*X*, Δ*t*), Fig 5(C) and 5(E). Thus, the different initial conditions are equivalent to different realizations of dynamic noise that drives diffusive motion along the approximate line attractor.

### Additional randomness in connectivity and inputs

In addition to the results described above, we investigate several scenarios in which we introduce additional randomness, either frozen or dynamic. First, we relax the assumption that connections in the two sub-networks precisely mirror each other. This assumption was made above for convenience: the precise identity of the synaptic connections simplifies the numerical analysis since it ensures a precise symmetry of the dynamics around the hyperplane *X* = 0. S2 Fig demonstrates that the main conclusions of our analysis remain valid when the connectivity in each sub-network is drawn independently: dynamics are slow along the approximate line attractor, and the diffusion coefficient along the line scales as 1/*N* with a prefactor which is somewhat larger than the value observed in Fig 5.

The main new feature that arises when the synaptic connections are drawn independently in the two sub-networks, is that the relaxation point of the dynamics along the approximate attractor deviates from the hyperplane *X* = 0. The characteristic magnitude of this deviation decays monotonically to zero with increase of the system size *N*. We note that the mean field description of the dynamics for finite *K* ≫ 1 and in the limit *N* → ∞, is identical to the mean field dynamics associated with the perfectly symmetric scenario.

Next, we consider a scenario in which the inhibitory connections between the two sub-networks are random, and follow the same basic architecture as the connections within each sub-network. Therefore, instead of assuming weak all-to-all connections of order $\sqrt{K}/N$, we include random connections of order $1/\sqrt{K}$, with a probability *K/N* for a synaptic connection. In addition, we relax the assumption of mirrored connections in the two sub-networks. In this case it is straightforward to show that the mean-field equations remain identical to those associated with the case of all-to-all connections, in the limit *N* → ∞, *K* → ∞. Therefore, a continuous set of balanced states can be achieved (Methods).

When *K* is large and finite, the mean-field equations are slightly different in the two scenarios (Methods). The main outcome of this difference is a shift of the unstable fixed points of the dynamics from the planes *m*_1_ = *m*_2_ = 0 and *m*_3_ = *m*_4_ = 0. Consequently, there is a certain degree of activity in both sub-networks even in the unstable fixed points of the dynamics. This is shown in S3 Fig More significantly, S3 Fig demonstrates that in the case of random and sparse connections between the two sub-networks, the dynamics exhibit the same characteristics as in the case of all-to-all connectivity, and can be accurately approximated as an OU process over time scales longer than *τ*. The coefficient of diffusion *D* scales linearly with 1/*N*, with a prefactor which is close to the one observed in S2 Fig.

Finally, we explore the effects of stochasticity in the input *E*_0_ to the network (see Fig 1B). S4 Fig demonstrates that even when the inputs include a large degree of temporal variability, and the noise injected to all the neurons in the network is highly correlated, the network exhibits slow dynamics along an approximate attractor, with statistics that are qualitatively similar to what we present above.

## Discussion

In summary, we demonstrated that a simple balanced network can exhibit slow dynamics along a continuous line in the population mean activity space. In finite networks, the chaotic dynamics drive diffusive motion along the approximate line attractor. We calculated the diffusivity in the system, based on the correlation structure observed in a single balanced network, and showed that the diffusion coefficient along the approximate attractor is inversely proportional to the network size. This is similar to the effect of noise that arises from intrinsic neural or synaptic mechanisms [19].

The slow diffusive motion along a one-dimensional trajectory induces correlations within the populations, and in single neuron pairs, that persist up to a long time scale set by the decay time *λ*^−1^. This property characterizes the dynamics of the system even when it is at the resting state (*X* ≃ 0), *i.e.*, when the network is not engaged in a memory task. Hence, this observation generates a prediction for spontaneous activity in brain areas such as the prefrontal cortex, in which continuous attractor dynamics, based on mutual inhibition between two populations, have been postulated [33].

A slowly decaying cross-correlation function characterizes the spikes produced by any pair of neurons in the network, but due to the high irregularity in the single neuron activity it may be necessary to average over multiple simultaneously recorded neuron pairs in order to obtain a clear measurement over a realistic time scale for a single experiment (in Fig 7C, 50 neuron pairs and a ∼15 minute measurement). Furthermore, it is important to label the neurons based on their functional properties: averaged over all populations, the cross correlations seen in Fig 7A cancel. In brain areas involved in short term memory tasks, this labeling can potentially be achieved by first measuring the tuning curves of neurons as a function of the stored variable.

### Linear *vs*. nonlinear neural response in decision making circuits

Several models of decision making circuits in the prefrontal cortex were based on the simple neural architecture of Fig 1A [32]. This network architecture can precisely generate a continuous attractor if the activity of single units is a linear function of their input. While the linear dynamics provide a simple intuition for the principles underlying continuous attractor dynamics in recurrent neural networks [31], it is more difficult to obtain a continuum of steady states using the above network architecture when single neural responses are nonlinear. Therefore, specifically tuned forms of nonlinearity [33, 35], or more elaborate network architectures—still based on mutual inhibition between two or more neural populations have been proposed [33, 34]. In this context the linear input-output relationship, characterizing the single balanced network of Ref. [3], is a useful computational feature that facilitates the construction of a continuous attractor network based on the simple architecture of Fig 1A. However, the main motivation for considering the balanced state in this work lies in its ability to account for the irregular spiking of single neurons in cortical circuits.

### Time scales of persistence and retention of information

Continuous attractor networks are an important model for maintenance of short-term memory in the brain. The memory is represented by the position along the attractor, and therefore the stochastic motion along the attractor determines the fidelity of memory retention. Since the dynamics of our proposed network are well characterized as an OU process over time scales longer than *τ* and over a large range of positions along the approximate attractor, it is straightforward to assess how the position along the approximate attractor evolves in time. All aspects of the trajectory can be easily inferred based on the initial state along the approximate attractor, the time interval, and the two parameters which characterize the OU process: *λ* and *D*. Similar considerations can be applied also for noisy continuous attractors in which the stochasticity arises from mechanisms other than the chaotic dynamics studied here [19, 20]. We next discuss how the decay time and the diffusivity depend on the parameters of our model.

The decay time *λ*^−1^ can be calculated exactly in the limit of *N* → ∞, *K* ≫ 1 (Fig 2). It is interesting to note that there are competing influences of *K* on the tuning of the attractor: with increase of *K*, *λ*^−1^ becomes more sensitive to $\tilde{J}$. However, when *K* is reduced, the nullclines (Fig 2) become less linear, causing deviations from the ideal behavior far from the symmetry point. We showed that for *K* ∼ 10^3^ and *τ* = 10ms it is possible to achieve persistence over several seconds, while the decay time and diffusion coefficient are approximately constant along a wide range of positions. This requires to tune $\tilde{J}$ to a relative precision of order 0.1%.

Since all times scale linearly with the intrinsic time constant, longer persistence times (or a weaker tuning requirement) can be achieved if the intrinsic time constants of individual units is longer that the value of 10 ms assumed in our examples. Intrinsic neural persistence or slow synapses could potentially contribute to this goal under more realistic biophysical descriptions of the neural dynamics.

Finally, we note that the requirement for precise tuning of the connectivity is a characteristic feature of all continuous attractor models. Several works have proposed ways to achieve tuning through plasticity mechanisms [37, 38], or ways to stabilize the dynamics by additional mechanisms such as synaptic adaptation [28, 39, 40] or negative derivative feedback [29, 30], in order to increase the persistence time.

### Diffusive motion

The diffusive motion along the approximate attractor, which is the main focus of this work, poses an additional limitation on the persistence of short term memory. While appropriate readout mechanisms may be able to take into account the systematic drift caused by the decay towards to the symmetry point, random diffusion inherently degrades the information stored in the position along the attractor.

With 10^5^ neurons per population, random diffusion over an interval of one second causes a deflection in *X* with a standard deviation of ∼10^−2^. This quantity should be compared with the possible range of *X*, which is approximately [-0.2, 0.2] in our parametrization of the position along the approximate attractor (we verified that the dynamics are accurately approximated as an OU process over the range [-0.1, 0.1]). Therefore, with the tuning chosen in Fig 4C, where *λ*^−1^ ∼ 10s, and with *N* = 10^5^, the limiting factor for discrimination between nearby stimuli after a delay period of order 1 s is the diffusive dynamics along the approximate attractor.

Random diffusion in continuous attractor networks of Poisson neurons is also often very significant [19, 20]. The diffusivity can be suppressed by increasing the number of neurons, increasing the intrinsic time constant of individual neurons and synapses, or by assuming that the firing of individual neurons is sub-Poisson [19, 41]. Our proposed model for a line of persistent balanced states similarly predicts a significant degree of random diffusion, highlighting the need to better understand how noise influences the retention of continuous parameter memory in cortical circuits.

There are several ways in which the random, diffusive component of the motion can potentially be reduced: First, by increasing the number of neurons. We presented results for networks containing (altogether) up to 6 × 10^5^ neurons, and it is straightforward to extrapolate our estimates for *D* to larger networks based on the 1/*N* dependence of the diffusion coefficient. Second, the diffusion coefficient is expected to decrease significantly if slow synapses participate in the dynamics [19], or if the intrinsic time constant of the neurons is increased. Third, additional mechanisms such as synaptic adaptation [28, 40] or derivative feedback [29] may perhaps contribute to a reduction in the diffusivity. Finally, an intriguing possibility is that highly structured and tuned connectivity can yield improved robustness to noise in a balanced state, as hinted by recent results on predictive coding in spiking neural networks [42].

## Methods

### Model

In our model, two balanced neural subnetworks inhibit each other reciprocally: the inhibitory population in each subnetwork projects to the excitatory population of the other subnetwork, Fig 1B.

As in Refs. [3, 7], the neurons are binary and are updated asynchronously, at update times that follow Poisson statistics. The mean time interval between updates is *τ*_*E*_ (*τ*_*I*_) for neurons in the excitatory (inhibitory) populations. In each update of a neuron *k* from population *i*, the new state of the neuron $\sigma_{i}^{k}$ is determined based on the total weighted input to the neuron,

$$\begin{array}{c} \sigma_{i}^{k}=\Theta \left(u_{i}^{k}\right)\;, \end{array}$$ (8)

where Θ is the Heaviside step function, and $u_{i}^{k}$ is the total input to the unit at that time,

$$\begin{array}{c} u_{i}^{k}=\sum_{l=1}^{4}\left[\sum_{j=1}^{N_{l}}J_{kl}^{ij}\sigma_{l}^{j}\left(t\right)+\sqrt{K}E_{0}^{l}\right]-T_{k}\;. \end{array}$$ (9)

Here, *T*_*k*_ is the threshold and *E*_0_ is an external input. We chose the external input to be zero for the inhibitory populations and to be positive (and constant) for the excitatory populations. Connections within each network are random with a connection probability *K*/*N*, where 1 ≪ *K* ≪ *N*. Here *N* is the population size (chosen to be equal in all populations for simplicity) and *K* is the average number of inputs a neuron gets from each population. Connection strengths are: $J_{EE}/\sqrt{K},\;J_{IE}/\sqrt{K},\;J_{EI}/\sqrt{K}$ and $J_{II}/\sqrt{K}$ according to the identity of the participating neurons. Without loss of generality, we have chosen *J*_*EE*_ = *J*_*IE*_ = 1 and defined *J*_*EI*_ ≡ −*J*_*E*_, *J*_*II*_ ≡ −*J*_*I*_. Mutual inhibition is generated either by weak all-to-all connections (in all figures except for S3 Fig), or by strong random and sparse connections (S3 Fig). In the former scenario, synapses of strength $-\tilde{J}\sqrt{K}/N$ connect each inhibitory neuron to all excitatory neurons in the excitatory population of the other subnetwork. In the latter scenario (S3 Fig), connections from each inhibitory population to the excitatory population of the other subnetwork are chosen randomly with a connection probability *K*/*N*, and with strength $-\tilde{J}/\sqrt{K}$. An excitatory feed-forward input $\sqrt{K}E_{0}$ is fed into both excitatory populations. We denote by *u*_*i*_ the mean of $u_{i}^{k}$ over all the neurons *k* within the population *i* and over realizations of the connectivity:

$$\begin{array}{c} \begin{array}{ccc} u_{1} & = & \sqrt{K}\left(m_{1}-J_{E}m_{2}-\tilde{J}m_{4}+E_{0}\right)-T_{1}\;,\\ u_{2} & = & \sqrt{K}\left(m_{1}-J_{I}m_{2}\right)-T_{2}\;,\\ u_{3} & = & \sqrt{K}\left(m_{3}-J_{E}m_{4}-\tilde{J}m_{2}+E_{0}\right)-T_{3}\;,\\ u_{4} & = & \sqrt{K}\left(m_{3}-J_{I}m_{4}\right)-T_{4}\;. \end{array} \end{array}$$ (10)

Here *m*_*i*_ are the population averaged activities. The variance of $u_{i}^{k}$ over all the neurons *k* within the population *i* and over realizations of the connectivity is given (to leading order in *K*/*N*) by:

$$\begin{array}{c} \begin{array}{ccc} \alpha_{1} & = & m_{1}+J_{E}^{2}m_{2}\;,\\ \alpha_{2} & = & m_{1}+J_{I}^{2}m_{2}\;,\\ \alpha_{3} & = & m_{3}+J_{E}^{2}m_{4}\;,\\ \alpha_{4} & = & m_{3}+J_{I}^{2}m_{4}\;. \end{array} \end{array}$$ (11)

These expressions are obtained in similarity to the derivation of the variances in Ref. [7]. Note that the all-to-all inhibitory connections between the subnetworks contribute only terms of higher order in *K*/*N*. In the scenario where the connections between subnetworks are randomly drawn (S3 Fig), the variance of the input to the excitatory neurons includes an additional term, due to the variability of inhibitory synapses from the opposing sub-network. In this scenario

$$\begin{array}{c} \begin{array}{ccc} \alpha_{1} & = & m_{1}+J_{E}^{2}m_{2}+{\tilde{J}}^{2}m_{4}\;,\\ \alpha_{2} & = & m_{1}+J_{I}^{2}m_{2}\;,\\ \alpha_{3} & = & m_{3}+J_{E}^{2}m_{4}+{\tilde{J}}^{2}m_{2}\;,\\ \alpha_{4} & = & m_{3}+J_{I}^{2}m_{4}\;. \end{array} \end{array}$$ (12)

The mean field equations written below are valid both for all-to-all and for random connections between sub-networks, with the appropriate choice of *α*_*i*_.

#### Line of balanced states in the limit *N* ≫ *K* ≫ 1

To check whether there exist parameters for which the system has a continuum of balanced states, it is convenient to write the steady state of Eq (3) as follows:

$$\begin{array}{c} \begin{array}{ccc} m_{1}-J_{E}m_{2}-\tilde{J}m_{4}+E_{0} & = & \frac{1}{\sqrt{K}}\left[T_{1}-\sqrt{\alpha_{1}}H^{-1}\left(m_{1}\right)\right]\;,\\ m_{1}-J_{I}m_{2} & = & \frac{1}{\sqrt{K}}\left[T_{2}-\sqrt{\alpha_{2}}H^{-1}\left(m_{2}\right)\right]\;,\\ m_{3}-J_{E}m_{4}-\tilde{J}m_{2}+E_{0} & = & \frac{1}{\sqrt{K}}\left[T_{3}-\sqrt{\alpha_{3}}H^{-1}\left(m_{3}\right)\right]\;,\\ m_{3}-J_{I}m_{4} & = & \frac{1}{\sqrt{K}}\left[T_{4}-\sqrt{\alpha_{4}}H^{-1}\left(m_{4}\right)\right]\;. \end{array} \end{array}$$ (13)

Taking the limit *K* → ∞ while requiring that none of the populations is fully on or off produces a linear system of equations for the mean activities, Eq 4. When $\tilde{J}=J_{E}-J_{I}$ the system of linear equations is singular. In this case the steady state equations admit a continuum of solutions which comprise a continuum of stable balanced states. A possible parametrization of the line of balanced states is given by:

$$\begin{array}{ccc} m_{1} & = & x\;,\\ m_{2} & = & x/J_{I}\;,\\ m_{3} & = & -x+J_{I}E_{0}/\left(J_{E}-J_{I}\right)\;,\\ m_{4} & = & -x/J_{I}+E_{0}/\left(J_{E}-J_{I}\right)\;. \end{array}$$ (14)

The conditions *J*_*E*_ − *J*_*I*_ > 0, *J*_*I*_ > 1, and 0 < *J*_*I*_ *E*_0_/(*J*_*E*_ − *J*_*I*_) < 1 ensure that for 0 < *x* < *J*_*I*_ *E*_0_/(*J*_*E*_ − *J*_*I*_) the mean activities are positive and none of them is equal to 0 or 1.

In Fig 2(E) and 2(F), we artificially add to the dynamics (Eq 3) white Gaussian noise as follows:

$$\begin{array}{c} \tau_{i}{\dot{m}}_{i}=-m_{i}+H\left(u_{i}/\sqrt{\alpha_{i}}\right)+\xi_{i} \end{array}$$ (15)

where 〈*ξ*_*i*_(*t*)〉 = 0 and

$$\begin{array}{c} \left\langle \xi_{i}\left(t\right)\xi_{j}\left(t^{\prime }\right)\right\rangle =\sigma^{2}\delta_{ij}\delta \left(t-t^{\prime }\right)\;. \end{array}$$ (16)

Note that this was done only in Fig 2(E) and 2(F), to illustrate the existence of slow dynamics along a line in the case of infinite *N*. There is no injected noise elsewhere, and in particular there is no injected noise in our simulations of finite *N* networks.

### Simulations and statistics of the diffusive dynamics

Our results for networks with finite *N* are based on large scale numerical simulations. In each simulation the connections were chosen randomly as described in the text, and an asynchronous update schedule was generated by a Poisson process. Parameter values are specified in the legend of Fig 2 in the main text. Averaged population activities were calculated online. The projection along the approximate attractor was defined at each time point as

$$\begin{array}{c} X\left(t\right)\equiv v_{0}^{T}\cdot \left[m\left(t\right)-m_{0}\right]\;, \end{array}$$ (17)

where ***m***(*t*) is the measured 4 dimensional averaged population activity, ***m***_0_ is the vector of mean population activities at the symmetric fixed point, and ***v***_0_ is the left eigenvector of the linearized dynamics with an eigenvalue close to zero. We chose the following normalization for the corresponding right eigenvector (see Eq 14):

$$\begin{array}{c} \left(\begin{array}{c} 1\\ 1/J_{I}\\ -1\\ -1/J_{I} \end{array}\right)\;, \end{array}$$ (18)

and the normalization of *v*_0_ was chosen such that the dot product of the left eigenvector and the right eigenvector equals unity.

Measurements of *G*(*X*, Δ*t*) (Eq 6) were done in the following way: for each value of *X* we found all the time points for which |*X*(*t*_*i*_) − *X*| < *δ*, using a small *δ* ≃ 10^−3^. Then, for each such *t*_*i*_ we calculated [*X*(*t*_*i*_ + Δ*t*) − *X*(*t*_*i*_)]^2^, and averaged all these values to get *G*(*X*, Δ*t*). Subsequently, we averaged over multiple simulations with different quenched noise and update schedules. In the manuscript we present results for *G*(0, Δ*t*), but in similarity to *F*(*X*, Δ*t*)/*X*, *G*(*X*, Δ*t*) was fairly uniform along the approximate attractor. A similar calculation was performed to measure the drift *F*(*X*, Δ*t*). In Fig 5(C)–5(F), results are based on (1−2) × 10^3^ simulations with random initial conditions, each spanning a simulated time of about 10 seconds. In simulations of the finite *N* network, we estimated *λ* from measurements of *F*(*X*, Δ*t*) near the symmetric fixed point, and tuned $\tilde{J}$ to obtain *λ*^−1^ ≫ *τ*. In Fig 5, *λ*^−1^ ≃ 2 seconds.

#### Measurement of cross covariance functions

The cross covariance functions shown in Fig 7 were calculated in the following way: first, we measured the activity of *n* neurons at *M* equally spaced time points, with a time difference Δ*t* = 66ms. Then, for each pair of neurons *i*, *j* of populations *k*, *l* respectively, we calculated the unbiased estimate of the cross covariance:

$$\begin{array}{c} C_{l,k}^{i,j}\left(t_{m}\right)=\frac{1}{M-|m|}\sum_{a=0}^{M-|m|-1}\sigma_{k}^{i}\left(t_{a+m}\right)\sigma_{l}^{j}\left(t_{a}\right)-\left[\frac{1}{M}\sum_{a=0}^{M-1}\sigma_{k}^{i}\left(t_{a}\right)\right]\left[\frac{1}{M}\sum_{a=0}^{M-1}\sigma_{l}^{j}\left(t_{a}\right)\right]\;, \end{array}$$ (19)

Here *t*_*a*_ = *a*Δ*t*. Now, we averaged over all the measured pairs:

$$\begin{array}{c} C_{l,k}\left(t_{m}\right)=\frac{1}{0.5n(n-1)}\sum_{i\neq j}C_{l,k}^{i,j}\left(t_{m}\right)\;. \end{array}$$ (20)

For the calculation of the entire population averaged cross covariance we used the measured mean activities:

$$\begin{array}{c} C_{l,k}\left(t_{m}\right)=\frac{1}{M-|m|}\sum_{a=0}^{M-|m|-1}m_{k}\left(t_{a+m}\right)m_{l}\left(t_{a}\right)-\left[\frac{1}{M}\sum_{a=0}^{M-1}m_{k}\left(t_{a}\right)\right]\left[\frac{1}{M}\sum_{a=0}^{M-1}m_{l}\left(t_{a}\right)\right]\;. \end{array}$$ (21)

where $m_{l}\left(t\right)=1/N\sum_{i=1}^{N}\sigma_{l}^{i}\left(t\right)$. Note that the sum in Eq 21 includes the auto-covariances, while the expression in Eq 20 does not. However, the contribution of the auto-covariances is negligible in the entire population average, since its contribution, relative to the contribution of cross-covariances scales as 1/*N*.

### Diffusion over short time scales

To analytically evaluate *G*(*X*, Δ*t*) (Eq 6) over short time scales, we start by writing the change in the state of the *k*-th neuron in population *i* in a short time interval Δ*t* as:

$$\begin{array}{c} \sigma_{i}^{k}\left(t+\Delta t\right)-\sigma_{i}^{k}\left(t\right)=c_{i}^{k}\left(t\right)\left[\Theta_{i}^{k}\left(t\right)-\sigma_{i}^{k}\left(t\right)\right]\;, \end{array}$$ (22)

where $\Theta_{i}^{k}\left(t\right)$ is the outcome of an update if it occurs, and $c_{i}^{k}\left(t\right)$ is a random variable equal to 1 if the *i*-th neuron was updated between *t* and *t* + Δ*t* and to 0 otherwise. The updates occur each *τ* ms on average, so that

$$\begin{array}{c} {\left\langle c_{i}^{k}\left(t\right)\right\rangle }_{t}={\left\langle c_{i}^{k}{\left(t\right)}^{2}\right\rangle }_{t}=\frac{\Delta t}{\tau_{k}}\;, \end{array}$$ (23)

whereas for *i* ≠ *j* and/or *k* ≠ *l*,

$$\begin{array}{c} {\left\langle c_{i}^{k}\left(t\right)c_{j}^{l}\left(t\right)\right\rangle }_{t}=\frac{{\left(\Delta t\right)}^{2}}{\tau_{k}\tau_{l}}\;. \end{array}$$ (24)

Now the mean squared displacement, *G*(*X*, Δ*t*), can be written as:

$$\begin{array}{c} \left\langle {\left[X(t+\Delta t)-X(t)\right]}^{2}\right\rangle =\frac{1}{N^{2}}\sum_{i,j=1}^{4}\sum_{k,l=1}^{N}v_{i}^{0}v_{j}^{0}\left\langle \left[\sigma_{i}^{k}\left(t+\Delta t\right)-\sigma_{i}^{k}\left(t\right)\right]\left[\sigma_{j}^{l}\left(t+\Delta t\right)-\sigma_{j}^{l}\left(t\right)\right]\right\rangle \;, \end{array}$$ (25)

where $v_{i}^{0}$ is the i’th component of the left eigenvector of the Jacobian with eigenvalue close to zero. From Eqs 23 and 24 we see that for Δ*t* ≪ *τ* the contribution of elements with *i* = *j*, *k* = *l* dominates the sum. To leading order in Δ*t* we have

$$\begin{array}{ccc} \left\langle {\left[X(t+\Delta t)-X(t)\right]}^{2}\right\rangle & ≃ & \frac{1}{N^{2}}\sum_{i=1}^{4}\sum_{k=1}^{N}{\left(v_{i}^{0}\right)}^{2}\\ & \times & \left\langle {\left[\sigma_{i}^{k}\left(t+\Delta t\right)-\sigma_{i}^{k}\left(t\right)\right]}^{2}\right\rangle \;. \end{array}$$ (26)

Defining

$$\begin{array}{c} q_{i}\left(\Delta t\right)=\frac{1}{N}\sum_{k=1}^{N}\left\langle \sigma_{i}^{k}\left(t+\Delta t\right)\sigma_{i}^{k}\left(t\right)\right\rangle \;, \end{array}$$ (27)

we obtain:

$$\begin{array}{c} G\left(X,\Delta t\right)≃\frac{2\Delta t}{N}\sum_{j=1}^{4}{\left(v_{j}^{0}\right)}^{2}{\left.\left(-\frac{\partial q_{j}\left(t\right)}{\partial t}\right)\right|}_{t\to 0}\;. \end{array}$$ (28)

### Diffusion over arbitrary time scales

To derive an expression for the diffusive dynamics over arbitrary time scales, we start by representing the stochastic linearized dynamics of a single balanced network, near the symmetric fixed point, as a two dimensional stochastic process:

$$\begin{array}{c} \begin{array}{c} \dot{\delta m}=B_{1}\delta m+B_{2}\delta E+\xi \;, \end{array} \end{array}$$ (29)

where ***δm*** is the deviation of the mean activities from the fixed point and ***δE*** is the deviation of the input from the constant input *E*_0_. Here *B*_1_ is a 2 × 2 matrix representing the response to perturbations in ***m***, and *B*_2_ is a 2 × 2 matrix representing the response to perturbations in the feedforward input. Both are obtained analytically from a linearization of the mean field dynamics. Finally, ***ξ*** is a random process with vanishing mean, whose covariance functions *C*_*ξ*_(Δ*t*) are stationary and are yet unspecified:

$$\begin{array}{c} C_{\xi ,ij}\left(t-t^{\prime }\right)\equiv \left\langle \xi_{i}\left(t\right)\xi_{j}\left(t^{\prime }\right)\right\rangle \;. \end{array}$$ (30)

Using Eq 29, it is straightforward to relate *C*_*ξ*_(*t*) to the covariance of the activities (while assuming constant feedforward input, $\delta \bar{E}=0$):

$$\begin{array}{ccc} C_{\xi }\left(t\right) & = & -\frac{d^{2}}{dt^{2}}C^{m}\left(t\right)+\frac{d}{dt}\left[C^{m}\left(t\right)B_{1}^{T}-B_{1}C^{m}\left(t\right)\right]\\ & + & B_{1}C^{m}\left(t\right)B_{1}^{T}\;. \end{array}$$ (31)

Using the measurements of *C*^*m*^ from simulations, we can thus obtain *C*^*ξ*^ numerically, using the above equation. In similarity to *C*^*m*^, *C*^*ξ*^ decays to zero over a time scale of order *τ*. Altogether, Eq 29 describes the stochastic dynamics of a single balanced network close to the balanced state, in response to small fluctuations ***δE*** in the feedforward inputs. In the two-subnetwork architecture, each subnetwork is coupled only to the mean activity of the other subnetwork, because of the all-to-all connectivity. More specifically, the mean activity of each subnetwork linearly modulates the external input to the excitatory population of the other subnetwork. Therefore, we can approximate the state of the 4-population network as a stochastic process with the following dynamics:

$$\begin{array}{c} \dot{\delta m}=A\delta m+\xi \;, \end{array}$$ (32)

where ***δm*** is now a 4 dimensional vector, whose first (last) two entries represent the state of the first (second) subnetwork, and *A* is the Jacobian of the full 4 dimensional dynamics around the fixed point (Eq 38), related in a simple manner also to the matrices *B*_1,2_ defined above. The correlation matrix of the 4 dimensional noise vector ***ξ*** is given by

$$\begin{array}{c} {\tilde{C}}_{\xi }=\left(\begin{array}{cc} C_{\xi } & 0\\ 0 & C_{\xi } \end{array}\right)\;, \end{array}$$ (33)

where *C*_*ξ*_ is the 2 × 2 noise correlation matrix Eq (30) evalulated for a single balanced network receiving fixed excitatory input, equal to the mean input to each subnetwork at the symmetric fixed point. Finally, we use this description of the dynamics to predict the statistics of diffusion along the line. We multiply Eq 32 from the left by *v*_0_, the eigenvector with the eigenvalue close to zero, which we denote by *λ* (note below that *λ* < 0):

$$\begin{array}{c} \dot{X}=\lambda X+v_{0}^{T}\cdot \xi \;. \end{array}$$ (34)

Here, $X=v_{0}^{T}\cdot \delta m$. Thus, we obtain using the Wiener—Khintchine theorem the time dependent correlation function of *X*,

$$\begin{array}{c} C_{X}\left(t\right)=-\frac{1}{2\lambda }\int_{-\infty }^{\infty }e^{\lambda |t^{\prime }|}v_{0}^{T}{\tilde{C}}_{\xi }\left(t-t^{\prime }\right)v_{0}dt^{\prime }\;. \end{array}$$ (35)

Finally, the diffusion over an arbitrary time interval Δ*t* is given by:

$$\begin{array}{c} \left\langle {\left[X(t+\Delta t)-X(t)\right]}^{2}\right\rangle =2\left[C_{X}\left(0\right)-C_{X}\left(\Delta t\right)\right]\;. \end{array}$$ (36)

### Proof: ∂*m*_1_/∂*m*_3_ = −1↔ vanishing eigenvalue

Here we show that when ∂*m*_1_/∂*m*_3_ = −1 at the symmetric point (*m*_1_ = *m*_3_, *m*_2_ = *m*_4_), the Jacobian matrix has a vanishing eigenvalue, leading to slow dynamics near the fixed point. We denote:

$$\begin{array}{c} f_{i,j}\equiv \frac{\partial H\left(-u_{i}/\sqrt{\alpha_{i}}\right)}{\partial m_{j}}\;. \end{array}$$ (37)

In terms of these quantities, the Jacobian matrix can be written as

$$\begin{array}{c} A\equiv \left(\begin{array}{cccc} f_{1,1}-1 & f_{1,2} & 0 & f_{1,4}\\ f_{2,1}/\tau & (f_{2,2}-1)/\tau & 0 & 0\\ 0 & f_{3,2} & f_{3,3}-1 & f_{3,4}\\ 0 & 0 & f_{4,3}/\tau & (f_{4,4}-1)/\tau \end{array}\right)\;. \end{array}$$ (38)

At the symmetric fixed point *f*_1,1_ = *f*_3,3_, *f*_2,2_ = *f*_4,4_, *f*_1,2_ = *f*_3,4_, *f*_1,4_ = *f*_3,2_, and *f*_2,1_ = *f*_4,3_. The Jacobian’s eigenvalues at that point are then:

$$\begin{array}{c} \begin{array}{c} \lambda_{\pm }^{\pm }=\frac{1}{2}\left[\left(f_{1,1}-1\right)+\frac{f_{2,2}-1}{\tau }\right]\\ \pm \frac{1}{2}\sqrt{{\left(\left(f_{1,1}-1\right)-\frac{f_{2,2}-1}{\tau }\right)}^{2}+\frac{4}{\tau }f_{1,2}f_{2,1}\pm \frac{4}{\tau }f_{1,4}f_{2,1}}\;. \end{array} \end{array}$$ (39)

Next, we approximate the derivative ∂*m*_1_/∂*m*_3_ at the symmetric point. We use a first order Taylor expansion of the mean field equations to get:

$$\begin{array}{ccc} \delta m_{1} & = & f_{1,1}\delta m_{1}+f_{1,2}\delta m_{2}+f_{1,4}\delta m_{4}\;,\\ \delta m_{2} & = & f_{2,1}\delta m_{1}+f_{2,2}\delta m_{2}\;,\\ \delta m_{3} & = & f_{1,4}\delta m_{2}+f_{1,1}\delta m_{3}+f_{1,2}\delta m_{4}\;,\\ \delta m_{4} & = & f_{2,2}\delta m_{4}+f_{2,1}\delta m_{3}\;. \end{array}$$ (40)

Here, *δm*_*i*_ are the small deviations from the symmetric fixed point. Using these equations we can write *δm*_2_ and *δm*_4_ as functions of *δm*_1_ and *δm*_3_:

$$\begin{array}{c} \delta m_{2}=\frac{f_{2,1}}{1-f_{2,2}}\delta m_{1}\;\;\;;\;\;\;\delta m_{4}=\frac{f_{2,1}}{1-f_{2,2}}\delta m_{3}\;. \end{array}$$ (41)

Plugging these expressions into the equation for *δm*_1_ yields an expression for *δm*_1_ as a function of *δm*_3_. The derivative is:

$$\begin{array}{c} \frac{\partial m_{1}}{\partial m_{3}}=\frac{f_{1,4}f_{2,1}}{f_{1,1}f_{2,2}-f_{1,2}f_{2,1}+1-\left(f_{1,1}+f_{2,2}\right)}\;. \end{array}$$ (42)

Note that ∂*m*_1_/∂*m*_3_ = ∂*m*_2_/∂*m*_4_. Equating this derivative to −1 (noting that in this case, ∂*m*_1_/∂*m*_3_ = ∂*m*_3_/∂*m*_1_) yields:

$$\begin{array}{c} f_{1,4}=\frac{f_{1,2}f_{2,1}-1+f_{1,1}+f_{2,2}-f_{1,1}f_{2,2}}{f_{2,1}}\;. \end{array}$$ (43)

By inserting *f*_1,4_ into Eq 38 we get $\lambda_{-}^{-}=0$.

## Supporting information

S1 Fig Mean squared displacement of location along the approximate attractor. (Same dataset as in Fig 4C in the main text.) **A**-**B** The mean squared displacement (MSD) of the location along the line for initial location *X*(0) = 0.05 (**A**) and *X*(0) = 0.08 (**B**) as a function of time. Error bars represent the standard deviation of the mean (black). Red: fit to an OU process. OU parameters from fit: *D* = 1.85 × 10^−6^(10 ms)^−1^, *λ* = 10^−3^(10 ms)^−1^ in both panels.
(TIF)

S2 Fig Non-mirrored connectivity. Results for a network in which the internal connectivity in each sub-network is drawn independently. **A** Population averaged activity projected onto the *m*_1_ − *m*_3_ plane. **B** Mean activities of the four populations: blue for one sub-network and red for the other. The higher activities are those of the excitatory populations (*m*_1_ and *m*_3_). **C** Projection along the approximate attractor. **D** *G*(*X* = 0, Δ*t*) *vs*. Δ*t* as measured from simulations (black). Error bars: standard deviation of the mean (blue). Red: fit to an OU process. Here *N* = 1.5 × 10^5^ (compare with Fig 5C in the main text). **E** Diffusion coefficient as a function of *N*, with fit to ∝ 1/*N* dependence in red (compare with Fig 5D in the main text). **F** Absolute distance of the activity from the symmetry plane *X* = 0, averaged over time and over connectivity instances, plotted *vs*. *N*. Red error bars represent the standard deviation of the mean. In all panels *K* = 1000 and *N* = 1.5 × 10^5^. Other parameters are as in Fig 2.
(TIF)

S3 FigNetwork with random and sparse inhibitory connections between the sub-networks.**A** Projections of the nullclines ${\dot{m}}_{1}=0$ (blue) and ${\dot{m}}_{3}=0$ (red) on the *m*_1_ − *m*_3_ plane, based on Eqs 13 and 12. Here *K* = 1000, $\tilde{J}=1.8$. Insets show a schematic illustration of the nullclines near the fixed points, in which the angle between the lines is amplified for clarity. **B** Population activities projected onto the *m*_1_ − *m*_3_ plane. **C** Mean activities of the four populations: blue for one subnetwork and red for the other. The higher activities are those of the excitatory populations (*m*_1_ and *m*_3_). **D** Projection along the approximate attractor. **E** *G*(*X* = 0, Δ*t*) *vs*. Δ*t* as measured from simulations (black, with std of the mean errorbars in blue) and a fit to an OU process (red). (Compare with Fig 5C in the main text.) **F** Diffusion coefficient as a function of *N*. Red: fit to ∝ 1/*N* dependence (compare with Fig 5D in the main text). Here *K* = 1000, *N* = 1.5 × 10^5^, $\tilde{J}\approx 1.76$, and all other parameters are as in Fig 2.(TIF)

S4 FigEffects of correlated input noise.Results from simulations, for a network in which dynamical noise is added to the input *E*_0_. The noise has a correlation time of 3*τ*, which ensures that the correlation across neurons is not averaged out due to the asynchronous updating. The noise is described by an OU process: $\tau_{noise}\dot{\xi }=-\xi +\sigma_{noise}\eta \left(t\right)$, where *τ*_*noise*_ = 30 ms, and *η*(*t*) is a gaussian white noise, and the value of *σ*_*noise*_ was varied to control the noise amplitude. **A** Mean activities of the four populations for *σ* = *E*_0_/3: blue for one subnetwork and red for the other. The higher activities are those of the excitatory populations (*m*_1_ and *m*_3_). **B** *G*(*X* = 0, Δ*t*) for *σ*_*noise*_ = 0 (black), *σ*_*noise*_ = *E*_0_/30 (green) and *σ*_*noise*_ = *E*_0_/3 (blue). Error bars represent the standard deviation of the mean. Red: fits to an OU process. **C** Mean square displacement (MSD) of the location along the line for initial location *X*(0) = 0.05. Colors are the same as in **B**. In this figure *K* = 500, *N* = 1.5 × 10^5^, $\tilde{J}≃1.77$.(TIF)

## References

[1] Mainen ZF, Sejnowski TJ. Reliability of spike timing in neocortical neurons. Science. 1995;268(5216):1503–1506. 10.1126/science.7770778 7770778

[2] Holt GR, Softky WR, Koch C, Douglas RJ. Comparison of discharge variability in vitro and in vivo in cat visual cortex neurons. Journal of Neurophysiology. 1996;75(5):1806–1814. 8734581 10.1152/jn.1996.75.5.1806

[3] van Vreeswijk C, Sompolinsky H. Chaos in neuronal networks with balanced excitatory and inhibitory activity. Science. 1996;274(5293):1724–1726. 10.1126/science.274.5293.1724 8939866

[4] Shadlen MN, Newsome WT. The variable discharge of cortical neurons: implications for connectivity, computation, and information coding. Journal of neuroscience. 1998;18(10):3870–3896. 9570816 10.1523/JNEUROSCI.18-10-03870.1998 PMC6793166

[5] Sompolinsky H, Crisanti A, Sommers HJ. Chaos in random neural networks. Physical Review Letters. 1988;61(3):259 10.1103/PhysRevLett.61.259 10039285

[6] Monteforte M, Wolf F. Dynamical Entropy Production in Spiking Neuron Networks in the Balanced State. Phys Rev Lett. 2010;105:268104 10.1103/PhysRevLett.105.268104 21231716

[7] van Vreeswijk C, Sompolinsky H. Chaotic balanced state in a model of cortical circuits. Neural computation. 1998;10(6):1321–1371. 10.1162/089976698300017214 9698348

[8] van Vreeswijk C, Sompolinsky H. Les Houches Lectures LXXX on Methods and models in neurophysics; 2005.

[9] Renart A, de la Rocha J, Bartho P, Hollender L, Parga N, Reyes A, et al The asynchronous state in cortical circuits. science. 2010;327(5965):587–590. 10.1126/science.1179850 20110507 PMC2861483

[10] Compte A, Constantinidis C, Tegnér J, Raghavachari S, Chafee MV, Goldman-Rakic PS, et al Temporally irregular mnemonic persistent activity in prefrontal neurons of monkeys during a delayed response task. Journal of neurophysiology. 2003;90(5):3441–3454. 10.1152/jn.00949.2002 12773500

[11] Wimmer K, Nykamp DQ, Constantinidis C, Compte A. Bump attractor dynamics in prefrontal cortex explains behavioral precision in spatial working memory. Nature neuroscience. 2014;17(3):431–439. 10.1038/nn.3645 24487232

[12] Robinson DA. Integrating with Neurons. Annual Review of Neuroscience. 1989;12(1):33–45. 10.1146/annurev.neuro.12.1.33 2648952

[13] Tsodyks MV, Sejnowski T. Rapid state switching in balanced cortical network models. Network: Computation in Neural Systems. 1995;6(2):111–124. 10.1088/0954-898X_6_2_001

[14] Zhang K. Representation of spatial orientation by the intrinsic dynamics of the head-direction cell ensemble: a theory. Journal of Neuroscience. 1996;16(6):2112–2126. 8604055 10.1523/JNEUROSCI.16-06-02112.1996 PMC6578512

[15] Seung HS. How the brain keeps the eyes still. Proceedings of the National Academy of Sciences. 1996;93(23):13339–13344. 10.1073/pnas.93.23.13339 PMC24094 8917592

[16] Compte A, Brunel N, Goldman-Rakic PS, Wang XJ. Synaptic mechanisms and network dynamics underlying spatial working memory in a cortical network model. Cerebral Cortex. 2000;10(9):910–923. 10.1093/cercor/10.9.910 10982751

[17] Renart A, Song P, Wang XJ. Robust spatial working memory through homeostatic synaptic scaling in heterogeneous cortical networks. Neuron. 2003;38(3):473–485. 10.1016/S0896-6273(03)00255-1 12741993

[18] Barak O, Tsodyks M. Working models of working memory. Current Opinion in Neurobiology. 2014;25:20–24. 10.1016/j.conb.2013.10.008 24709596

[19] Burak Y, Fiete IR. Fundamental limits on persistent activity in networks of noisy neurons. Proceedings of the National Academy of Sciences. 2012;109(43):17645–17650. 10.1073/pnas.1117386109 PMC3491496 23047704

[20] Kilpatrick ZP, Ermentrout B, Doiron B. Optimizing working memory with heterogeneity of recurrent cortical excitation. The Journal of Neuroscience. 2013;33(48):18999–19011. 10.1523/JNEUROSCI.1641-13.2013 24285904 PMC6618706

[21] Bays PM. Noise in neural populations accounts for errors in working memory. The Journal of Neuroscience. 2014;34(10):3632–3645. 10.1523/JNEUROSCI.3204-13.2014 24599462 PMC3942580

[22] Renart A, Moreno-Bote R, Wang XJ, Parga N. Mean-driven and fluctuation-driven persistent activity in recurrent networks. Neural computation. 2007;19(1):1–46. 10.1162/neco.2007.19.1.1 17134316

[23] Litwin-Kumar A, Doiron B. Slow dynamics and high variability in balanced cortical networks with clustered connections. Nature neuroscience. 2012;15(11):1498–1505. 10.1038/nn.3220 23001062 PMC4106684

[24] Rosenbaum R, Doiron B. Balanced networks of spiking neurons with spatially dependent recurrent connections. Physical Review X. 2014;4(2):021039 10.1103/PhysRevX.4.021039

[25] Stern M, Sompolinsky H, Abbott L. Dynamics of random neural networks with bistable units. Physical Review E. 2014;90(6):062710 10.1103/PhysRevE.90.062710 PMC4348075 25615132

[26] Roudi Y, Latham PE. A balanced memory network. PLoS Comput Biol. 2007;3(9):e141 10.1371/journal.pcbi.0030141 PMC1971123 17845070

[27] Hopfield JJ. Neural networks and physical systems with emergent collective computational abilities. Proceedings of the national academy of sciences. 1982;79(8):2554–2558. 10.1073/pnas.79.8.2554 PMC346238 6953413

[28] Hansel D, Mato G. Short-Term Plasticity Explains Irregular Persistent Activity in Working Memory Tasks. The Journal of Neuroscience. 2013;33(1):133–149. 10.1523/JNEUROSCI.3455-12.2013 23283328 PMC6618646

[29] Lim S, Goldman MS. Balanced cortical microcircuitry for maintaining information in working memory. Nature Neuroscience. 2013;16(9):1306–1314. 10.1038/nn.3492 23955560 PMC3772089

[30] Lim S, Goldman MS. Balanced Cortical Microcircuitry for Spatial Working Memory Based on Corrective Feedback Control. The Journal of Neuroscience. 2014;34(20):6790–6806. 10.1523/JNEUROSCI.4602-13.2014 24828633 PMC4019795

[31] Cannon SC, Robinson DA, Shamma S. A proposed neural network for the integrator of the oculomotor system. Biological cybernetics. 1983;49(2):127–136. 10.1007/BF00320393 6661444

[32] Wang XJ. Probabilistic decision making by slow reverberation in cortical circuits. Neuron. 2002;36(5):955–968. 10.1016/S0896-6273(02)01092-9 12467598

[33] Machens CK, Romo R, Brody CD. Flexible control of mutual inhibition: a neural model of two-interval discrimination. Science. 2005;307(5712):1121–1124. 10.1126/science.1104171 15718474

[34] Deco G, Rolls ET. Decision-making and Weber’s law: a neurophysiological model. European Journal of Neuroscience. 2006;24(3):901–916. 10.1111/j.1460-9568.2006.04940.x 16930418

[35] Polk A, Litwin-Kumar A, Doiron B. Correlated neural variability in persistent state networks. Proceedings of the National Academy of Sciences. 2012;109(16):6295–6300. 10.1073/pnas.1121274109 PMC3341079 22474377

[36] Funahashi S, Bruce CJ, Goldman-Rakic PS. Mnemonic coding of visual space in the monkey’s dorsolateral prefrontal cortex. Journal of neurophysiology. 1989;61(2):331–349. 2918358 10.1152/jn.1989.61.2.331

[37] Seung HS. Learning Continuous Attractors in Recurrent Networks In: NIPS. vol. 97 Citeseer; 1997 p. 654–660.

[38] MacNeil D, Eliasmith C. Fine-tuning and the stability of recurrent neural networks. PloS one. 2011;6(9):e22885 10.1371/journal.pone.0022885 21980334 PMC3181247

[39] Mongillo G, Barak O, Tsodyks M. Synaptic theory of working memory. Science. 2008;319(5869):1543–1546. 10.1126/science.1150769 18339943

[40] Itskov V, Hansel D, Tsodyks M. Short-term facilitation may stabilize parametric working memory trace. Frontiers in computational neuroscience. 2011;5:40 10.3389/fncom.2011.00040 22028690 PMC3199447

[41] Burak Y, Fiete IR. Accurate path integration in continuous attractor network models of grid cells. PLoS Comput Biol. 2009;5(2):e1000291 10.1371/journal.pcbi.1000291 19229307 PMC2632741

[42] Boerlin M, Machens CK, Denève S. Predictive coding of dynamical variables in balanced spiking networks. PLoS Comput Biol. 2013;9(11):e1003258 10.1371/journal.pcbi.1003258 24244113 PMC3828152

