# Supplementary figures and images for: Slow diffusive dynamics in a chaotic balanced neural network
Source: PLoS Comput Biol. 2017 May 1;13(5):e1005505. doi: 10.1371/journal.pcbi.1005505 (PMC5432195; doi:10.1371/journal.pcbi.1005505)

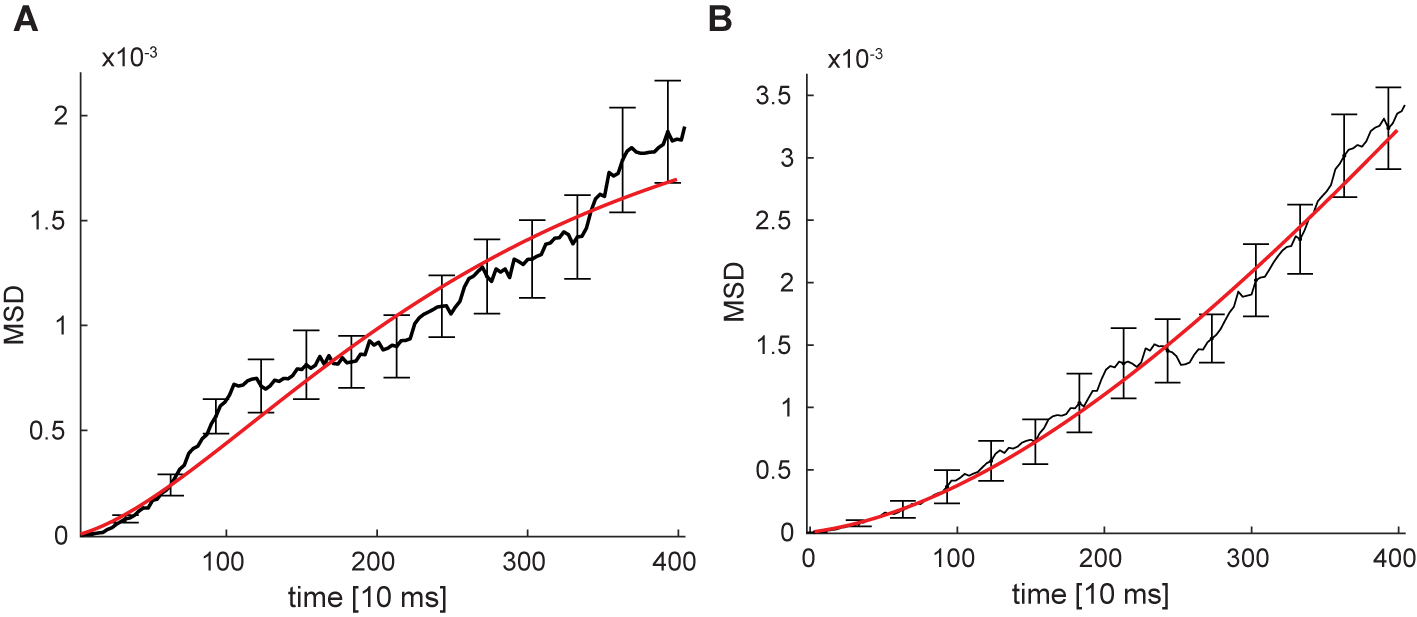

Supplement: S1 Fig — (Same dataset as in Fig 4C in the main text.) A-B The mean squared displacement (MSD) of the location along the line for initial location X(0) = 0.05 (A) and X(0) = 0.08 (B) as a function of time. Error bars represent the standard deviation of the mean (black). Red: fit to an OU process. OU parameters from fit: D = 1.85 × 10^−6^(10 ms)−1, λ = 10−3(10 ms)−1 in both panels. (TIF) [file pcbi.1005505.s001.tif]

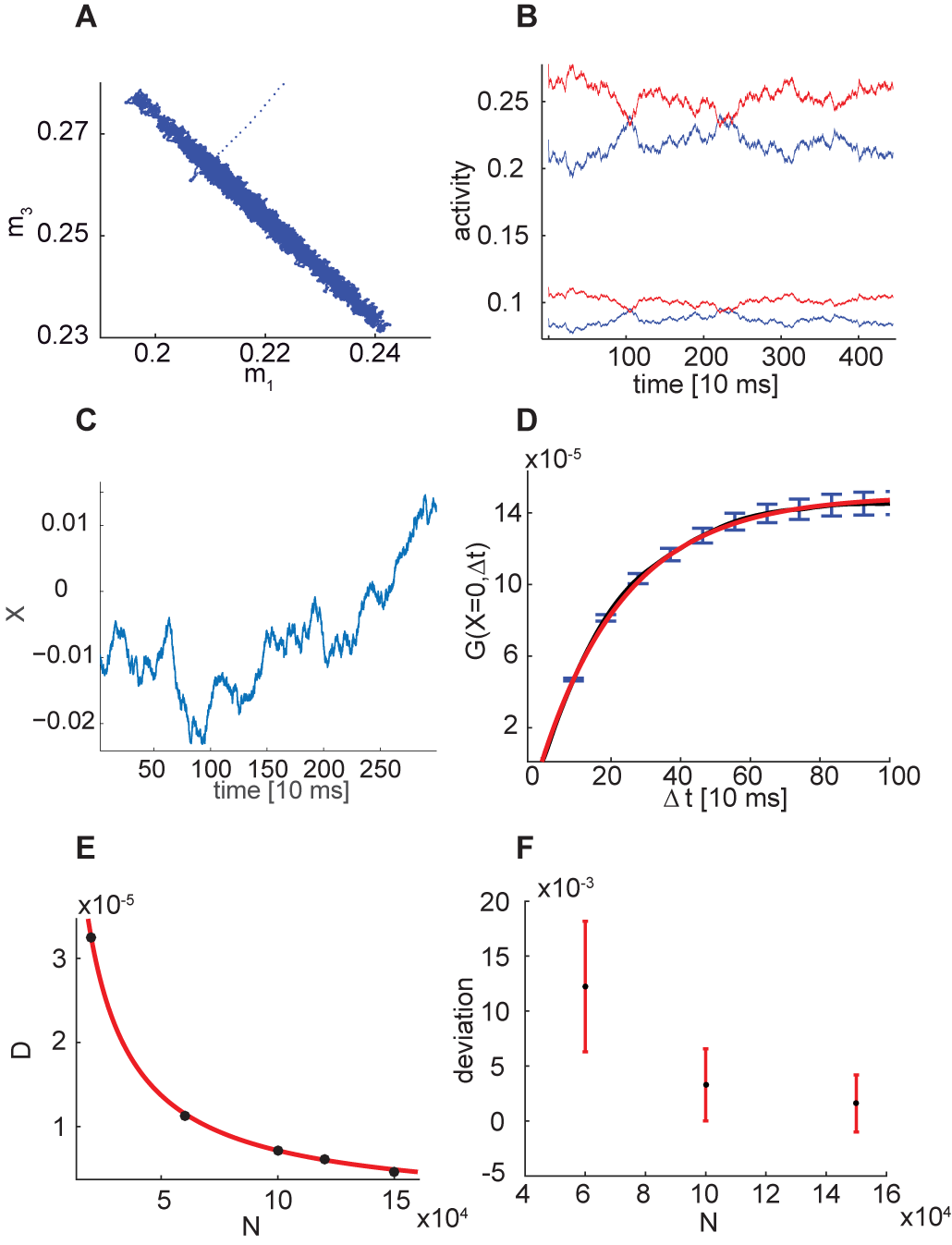

Supplement: S2 Fig — Results for a network in which the internal connectivity in each sub-network is drawn independently. A Population averaged activity projected onto the m1 − m3 plane. B Mean activities of the four populations: blue for one sub-network and red for the other. The higher activities are those of the excitatory populations (m1 and m3). C Projection along the approximate attractor. D G(X = 0, Δt) vs. Δt as measured from simulations (black). Error bars: standard deviation of the mean (blue). Red: fit to an OU process. Here N = 1.5 × 10^5^ (compare with Fig 5C in the main text). E Diffusion coefficient as a function of N, with fit to ∝ 1/N dependence in red (compare with Fig 5D in the main text). F Absolute distance of the activity from the symmetry plane X = 0, averaged over time and over connectivity instances, plotted vs. N. Red error bars represent the standard deviation of the mean. In all panels K = 1000 and N = 1.5 × 10^5^. Other parameters are as in Fig 2. (TIF) [file pcbi.1005505.s002.tif]

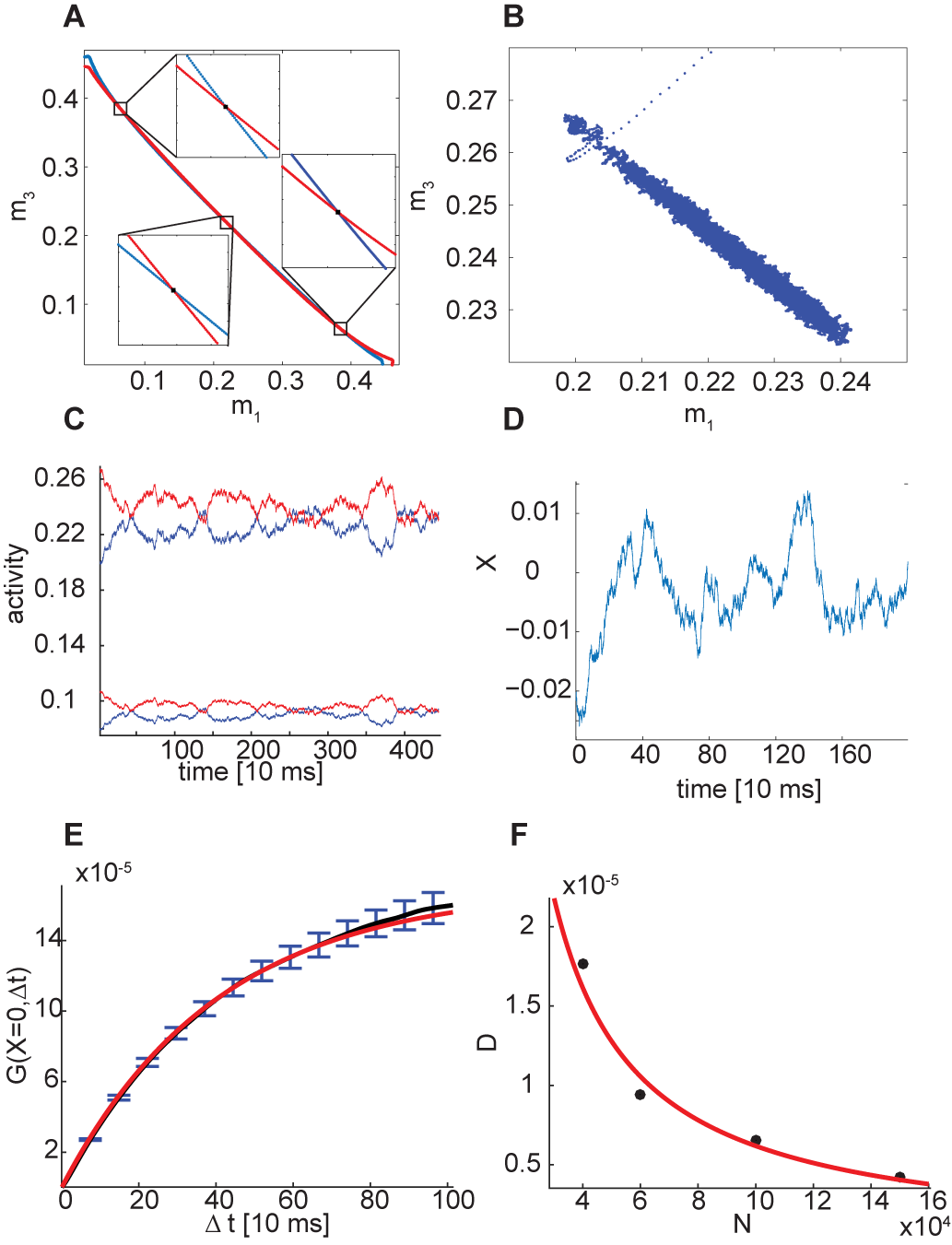

Supplement: S3 Fig — A Projections of the nullclines m˙1=0 (blue) and m˙3=0 (red) on the m1 − m3 plane, based on Eqs 13 and 12. Here K = 1000, J˜=1.8. Insets show a schematic illustration of the nullclines near the fixed points, in which the angle between the lines is amplified for clarity. B Population activities projected onto the m1 − m3 plane. C Mean activities of the four populations: blue for one subnetwork and red for the other. The higher activities are those of the excitatory populations (m1 and m3). D Projection along the approximate attractor. E G(X = 0, Δt) vs. Δt as measured from simulations (black, with std of the mean errorbars in blue) and a fit to an OU process (red). (Compare with Fig 5C in the main text.) F Diffusion coefficient as a function of N. Red: fit to ∝ 1/N dependence (compare with Fig 5D in the main text). Here K = 1000, N = 1.5 × 10^5^, J˜≈1.76, and all other parameters are as in Fig 2. (TIF) [file pcbi.1005505.s003.tif]

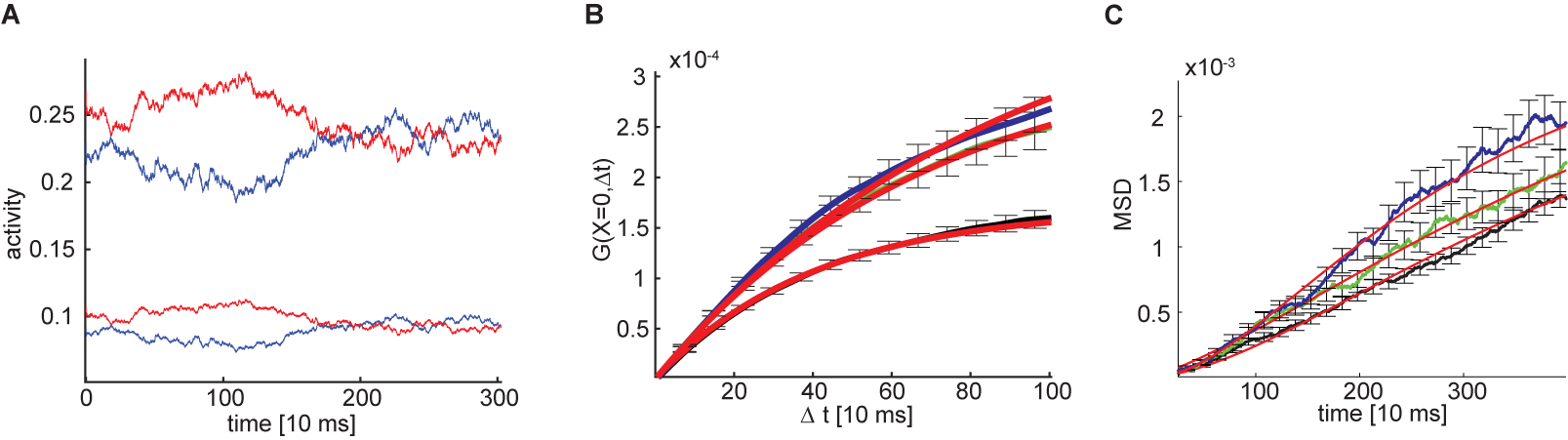

Supplement: S4 Fig — Results from simulations, for a network in which dynamical noise is added to the input E0. The noise has a correlation time of 3τ, which ensures that the correlation across neurons is not averaged out due to the asynchronous updating. The noise is described by an OU process: τnoiseξ˙=-ξ+σnoiseη(t), where τnoise = 30 ms, and η(t) is a gaussian white noise, and the value of σnoise was varied to control the noise amplitude. A Mean activities of the four populations for σ = E0/3: blue for one subnetwork and red for the other. The higher activities are those of the excitatory populations (m1 and m3). B G(X = 0, Δt) for σnoise = 0 (black), σnoise = E0/30 (green) and σnoise = E0/3 (blue). Error bars represent the standard deviation of the mean. Red: fits to an OU process. C Mean square displacement (MSD) of the location along the line for initial location X(0) = 0.05. Colors are the same as in B. In this figure K = 500, N = 1.5 × 10^5^, J˜≃1.77. (TIF) [file pcbi.1005505.s004.tif]
